# Natural talc: a basic, cost-effective, and available catalyst for the one-pot synthesis of dihydropyranochromenes and pyranopyrazoles, along with related DFT calculations

**DOI:** 10.1038/s41598-025-32187-4

**Published:** 2025-12-14

**Authors:** Abolfazl Dehghanizadeh, Bi Bi Fatemeh Mirjalili, Hadi Basharnavaz

**Affiliations:** Department of Chemistry, College of Science, Yazd University, Yazd, Iran

**Keywords:** Natural talc, Dihydropyranochromenes, Pyranopyrazoles, Mixer mill, DFT calculations, Chemistry, Materials science

## Abstract

This study investigates environmentally friendly and efficient protocols for synthesizing two distinct heterocyclic scaffolds: pyranopyrazoles and dihydropyranochromenes. Dihydropyranochromenes were synthesized using talc in refluxing ethanol, while pyranopyrazoles were obtained from solvent-free under mixing conditions. The advantages of these methods include high yields, operability, and the use of an accessible, inexpensive, and non-toxic basic catalyst. The reaction conditions, including reaction time, molar ratios of reactants, and catalyst amount, were optimized for the synthesis of products, resulting in high yields of 97% for pyranopyrazoles and 95% for dihydropyranochromenes. The synthesized products were characterized using analytical techniques, including FTIR and NMR, which confirmed the successful synthesis. In this study, total energy and band gap energy calculations were performed for dihydropyranochromenes and pyranopyrazoles derivatives. This suggests that talc powder a natural catalyst, can enhance organic synthesis and offer a sustainable and environmentally friendly solution for modern organic chemistry.

**Supplementary Information:**

The online version contains supplementary material available at 10.1038/s41598-025-32187-4.

## Introduction

 Heterocyclic compounds form the backbone of many pharmaceuticals, agrochemicals, and functional materials^1,2^. The development of efficient and sustainable synthetic routes for these compounds is of great interest. Multicomponent reactions (MCRs) have emerged as powerful tools in organic synthesis due to their atom economy, high efficiency, and the formation of complex molecules in a single step^3,4^. This study focuses on the synthesis of two important heterocyclic systems: dihydropyranochromenes and pyranopyrazoles, using talc as a catalyst under both solvent-and solvent-free conditions.

Dihydropyranochromenes are heterocyclic scaffolds with diverse biological activities in terms of their anticancer^5^, anti-inflammatory^6^, and antioxidant^7^ properties. Pyranopyrazoles are another class of heterocyclic compounds with biological activities^8^ and drug enhancers^9^ and have been used as antibacterial^10,11^, anticancer^12^, analgesic^13^, and antipyretic agents^14^. Given their importance, the development of efficient and environmentally friendly methods for their synthesis has become a vital research area.

Traditional methods for synthesizing pyranopyrazoles typically involve the use of solvents, extreme reaction conditions (such as high temperatures and pressures), and complex, costly catalysts, which contribute to significant environmental pollution^15–17^. These challenges underscore the necessity for sustainable and cost-effective alternatives that minimize environmental impacts while achieving high product yields. In response, a mechanochemical process using a mixer mill has been introduced as a green and efficient method for synthesizing pyranopyrazoles. This approach eliminates the need for organic solvents, allows the reactions to occur at ambient temperature and atmospheric pressure, reduces energy consumption, and facilitates easy control over the reaction conditions.

**Fig. 1:**
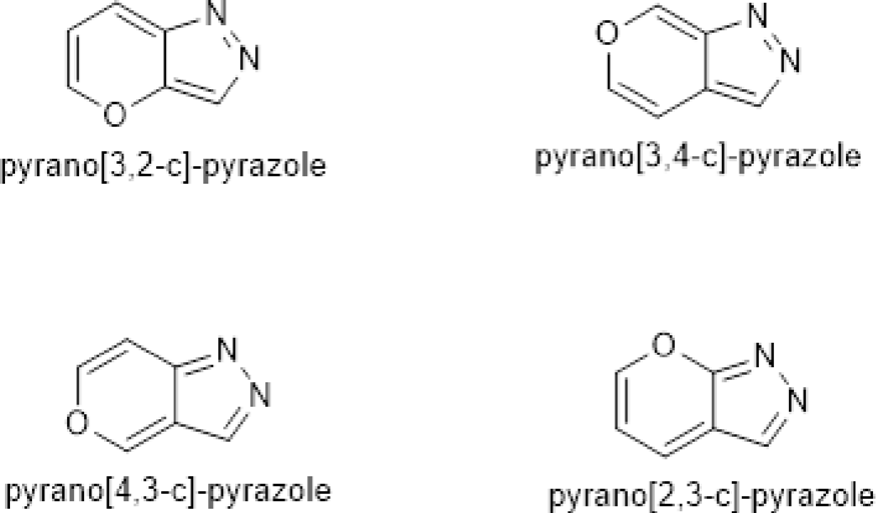
Four pyranopyrazole isomers.

Pyranopyrazole is a synthetic heterocyclic framework composed of pyran and pyrazole moieties. Pyranopyrazoles exist in four isomeric forms (Fig. 1). Among the four isomers, 4*H*-pyrano [2, 3-c]-pyrazole is the most privileged structuredue to its versatile biological profile.

Previously, several catalysts have been applied for the synthesis of pyranopyrazol such as, Fe_3_O_4_@SiO_2_@(CH_2_)_3_NH@CC@Imidazole@SO_3_H^18^, CaO@SiO_2_-SO_3_H^19^, Ru^III^@CMC/Fe_3_O_4_^20^, NiFe_2_O_4_@SiO_2_-H_14_[NaP_5_W_30_O_110_]^21^, Fe_3_O_4_@GO^22^, Fe_3_O_4_@THAM-SO_3_H^23^, H_14_ [NaP_5_W_30_O_110_]^24^.

Dihydropyranochromenes have been synthesized previously in the presence of sodium acetate^25^, DABCO^26^, zinc chloride^27^, SB-DABCO@eosin^28^, rGO@ Fe_3_O_4_^29^ and Silica-Bonded N-Propylpiperazine Sodium n-Propionate^30^.

Talс (Mg_3_Si_4_O_10_(OH)_2_)^31^, with Lewis base sites, is a green, available, low-cost catalyst. Talc can be used to promote base-catalyzed organic reactions such as tetrahydrobenzo[*b*]pyrans and benzo[*f*]chromen^31^. This study investigates the one-pot multicomponent synthesis of dihydropyranochromenes and pyranopyrazoles using talc powder as the catalyst. The objective of this study is to develop efficient, environmentally friendly, and economically viable synthesis routes for these significant heterocyclic compounds.

## Results and discussion

### Characterization of talk powder

FESEM shows the average particle size of the catalyst (506–759 nm).TGA analysis prove that talk is in thermally stable state. The percentage composition of O, Si, Mg, elements in talk is 54.10, 25.38 and 18.85 respectively which determined by EDX. a_s, BET_, V_p_ and pore diameter were 5.9994 m^2^ g^− 1^, 0.026818 cm^3^ g^− 1^, and 17.686 nm respectively^31^.

### Synthesis and characterization of Pyranopyrazoles under mixer milling condition

**Fig. 2:**
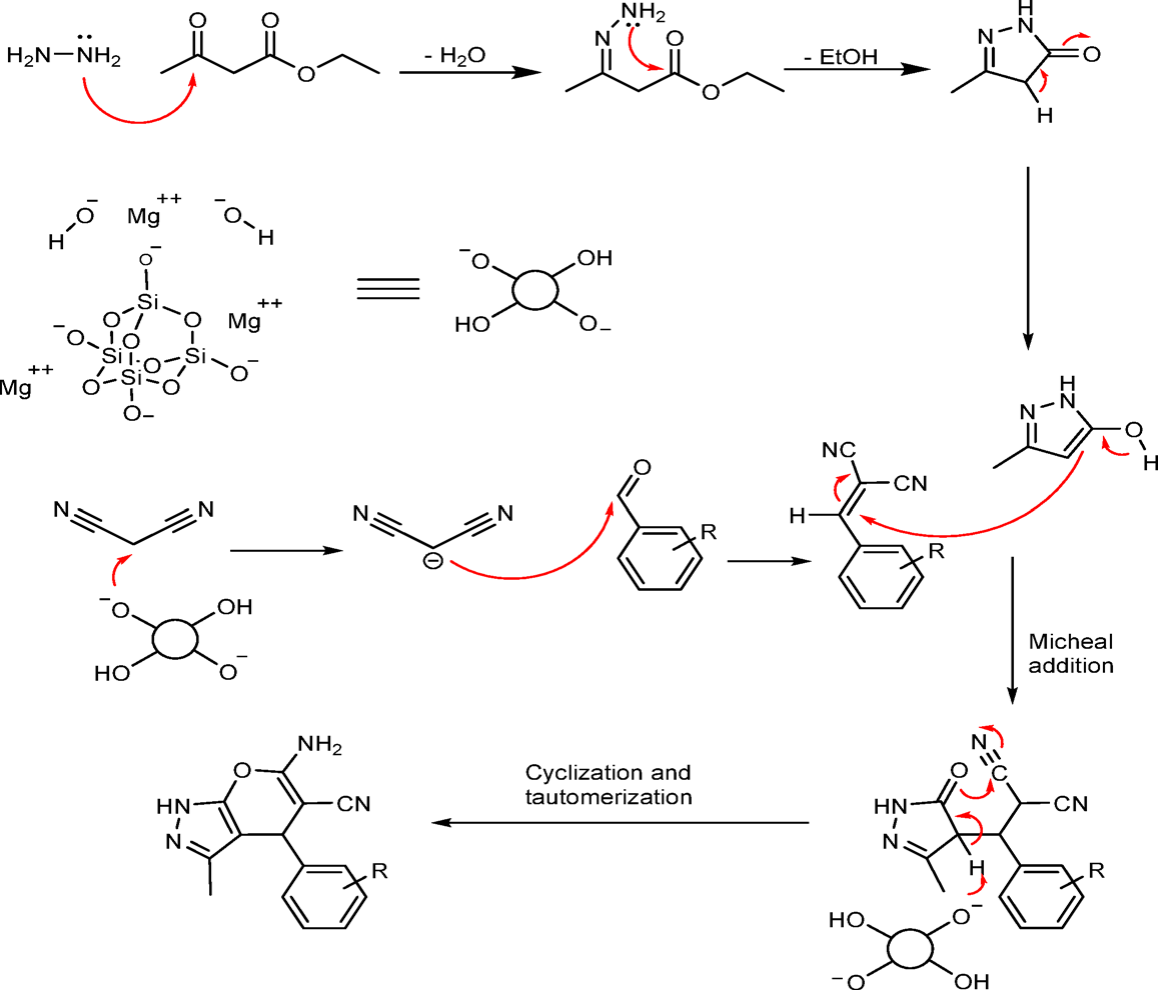
Proposed mechanism for the talc powder-catalyzed synthesis of pyranopyrazoles.

To evaluate the catalytic role of talc powder in the synthesis of pyranopyrazoles, the reaction was carried out in ethanol under reflux conditions as well as using a mixer mill at room temperature, both with and without the catalyst. The yield of pyranopyrazoles was 19% after 20 min for the reaction without a catalyst, while the talc powder-catalyzed experiment using a mixer mill achieved a 97% yield after just 5 min. This significant difference indicates the importance of a catalyst in the reaction steps. The proposed roles of the talc powder catalyst in the reaction mechanism are shown in Fig. 2.

**Fig. 3:**
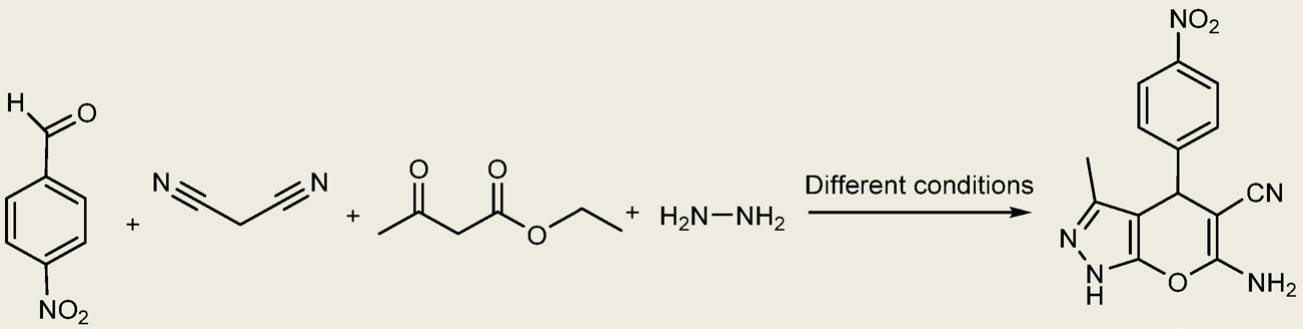
Synthesis of 6-Amino-3-methyl-4-(4-nitrophenyl)−1,4-dihydropyrano[2,3-c]pyrazole-5-carbonitrile under various condition (model reaction).

After the characterization of the basic talc powder, it was used for the synthesis of pyranopyrazoles. To optimize the reaction conditions, 4-nitrobenzaldehyde, hydrazine hydrate, ethyl acetoacetate, and malononitrile were used under different conditions, such as catalyst amount, temperature, and solvents (Fig. 3; Table 1).

**Fig. 4:**
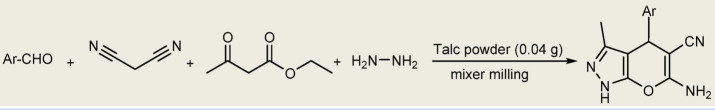
Synthesis of pyranopyrazoles under mixer milling condition.

Also, the model reaction was carried out in a stainless-steel vial and was conducted with two stainless-steel balls with a diameter of 0.8 mm at frequencies of 10, 15, and 20 Hz at room temperature using a mixer mill. At the end of the reaction, hot ethanol was added, and the entire reaction mixture was scraped; then the catalyst was separated. The progress of the reaction was monitored using thin-layer chromatography (TLC) and n-hexane, ethyl acetate (4:1) eluent. With the optimized reaction conditions (Table 1, entry 11), the best result was obtained using a mixer mill (frequency 20 Hz) and 0.04 g of catalyst without any solvent. The reaction conditions, including the molar ratio of the reactants, amount of catalyst, and milling time, were optimized to achieve the maximum yields (Fig. 4; Table 2).

**Table 1:** Comparative performances of the Talc catalyst for the synthesis of Pyranopyrazoles under different conditions.

Entry	Solvent/catalyst (g)	Condition	Time (min)	Yield (%)^a^
1	–/–	R.T.	120	Trace
2	H_2_O/–	R.T.	120	Trace
3	C_2_H_5_OH/–	R.T.	120	Trace
4	C_2_H_5_OH/–	Reflux	90	17
5	H_2_O/–	Reflux	90	5
6	C_2_H_5_OH/Talc (0.01)	Reflux	80	43
7	C_2_H_5_OH/Talc (0.02)	Reflux	80	51
8	C_2_H_5_OH/Talc (0.04)	Reflux	60	66
9	C_2_H_5_OH: H_2_O /Talc (0.04)	Reflux	60	44
10	–/Talc (0.04)	R.T.	60	71
11	–/Talc (0.04)	Mixer Mil 20 Hz	5	97
12	–/–	Mixer Mil 20 Hz	20	19
13	–/Talc (0.04)	Mixer Mil 15 Hz	15	71
14	–/Talc (0.04)	Mixer Mil 10 Hz	30	52
15	–/NaOH (0.04)	Mixer Mil 20 Hz	10	95^b^
16	–/K_2_CO_3_ (0.04)	Mixer Mil 20 Hz	15	90^b^
17	–/MgO (0.04)	Mixer Mil 20 Hz	10	95^b^
18	–/Hydrotalcite (0.04)	Mixer Mil 20 Hz	18	98^b^

The molar ratio of 4-nitrobenzaldehyde: malononitrile: ethylacetoacetate: hydrazine hydrate is 1 mmol:1.2 mmol: 1 mmol: 1.2 mmol.

^a^Isolated yield.

^b^Conversion yield.

**Table 2:** Synthesis of Pyranopyrazoles using mixer mill in the presence of Talc powder.

Compd.	Ar	Product	Time (min)	Yield (%)^b^	A.E.^c^ (%)	E.F.^d^	M.*P*. (°C)	M.*P*. (°C) (rep.)^Ref^.
1_a_	4-NO_2_-Ph		5	97	78	0.31	243–245	246–248^32^
2_a_	4-OH-Ph		10	91	80	0.43	221–224	222–224^32^
3_a_	4-Cl-Ph		5	92	77	0.4	236–238	235–237^33^
4_a_	2-OCH_3_-Ph		5	93	77	0.39	209–211	208–209^33^
5_a_	4-CH_3_-Ph		10	87	76	0.5	171–173	170–173^33^
6_a_	3,4-(OH)_2_-Ph		20	83	81	0.55	222–225	220–222^32^
7_a_	2,4-(Cl)_2_-Ph		15	85	83	0.41	223–226	223–225^33^
8_a_	3-OH,4-OCH_3_-Ph		15	88	78	0.45	233–235	234–236^32^
9_a_	2-Furyl		5	94	75	0.42	231–234	233–235^34^

^a^The molar ratio of aldehyde: malononitrile: hydrazine hydrate: ethylacetoacetate is 1mmol:1.2 mmol: 1.2 mmol:1 mmol.

^b^Isolated yield.

^c^Atomic Economy.

^d^E-Factor.

**Fig. 5:**
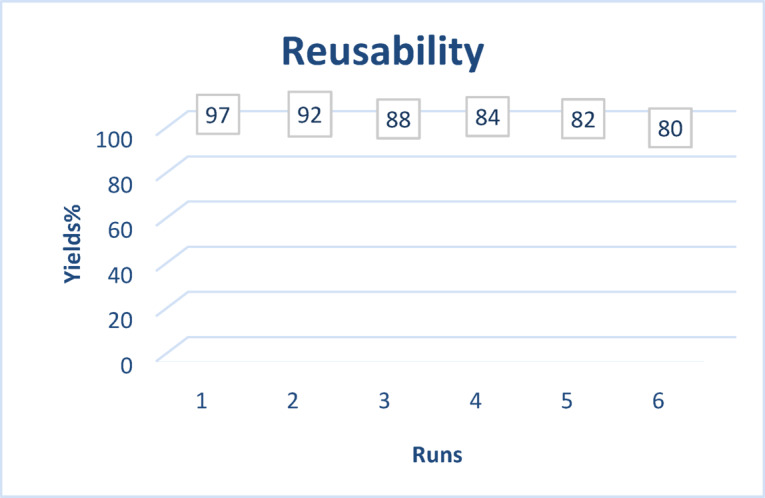
Reusability of Talc powder for the synthesis of pyranopyrazoles.

The reusability of catalyst for synthesis of pyranopyrazole, was investigated in model reaction for six runs. After each run, the catalyst was separated from the reaction mixture and washed with ethanol, dried in room temperature and reused in another run (Fig. 5).

### Synthesis and characterization of dihydropyrano[*3*,*2-c*]chromenes

**Fig. 6:**
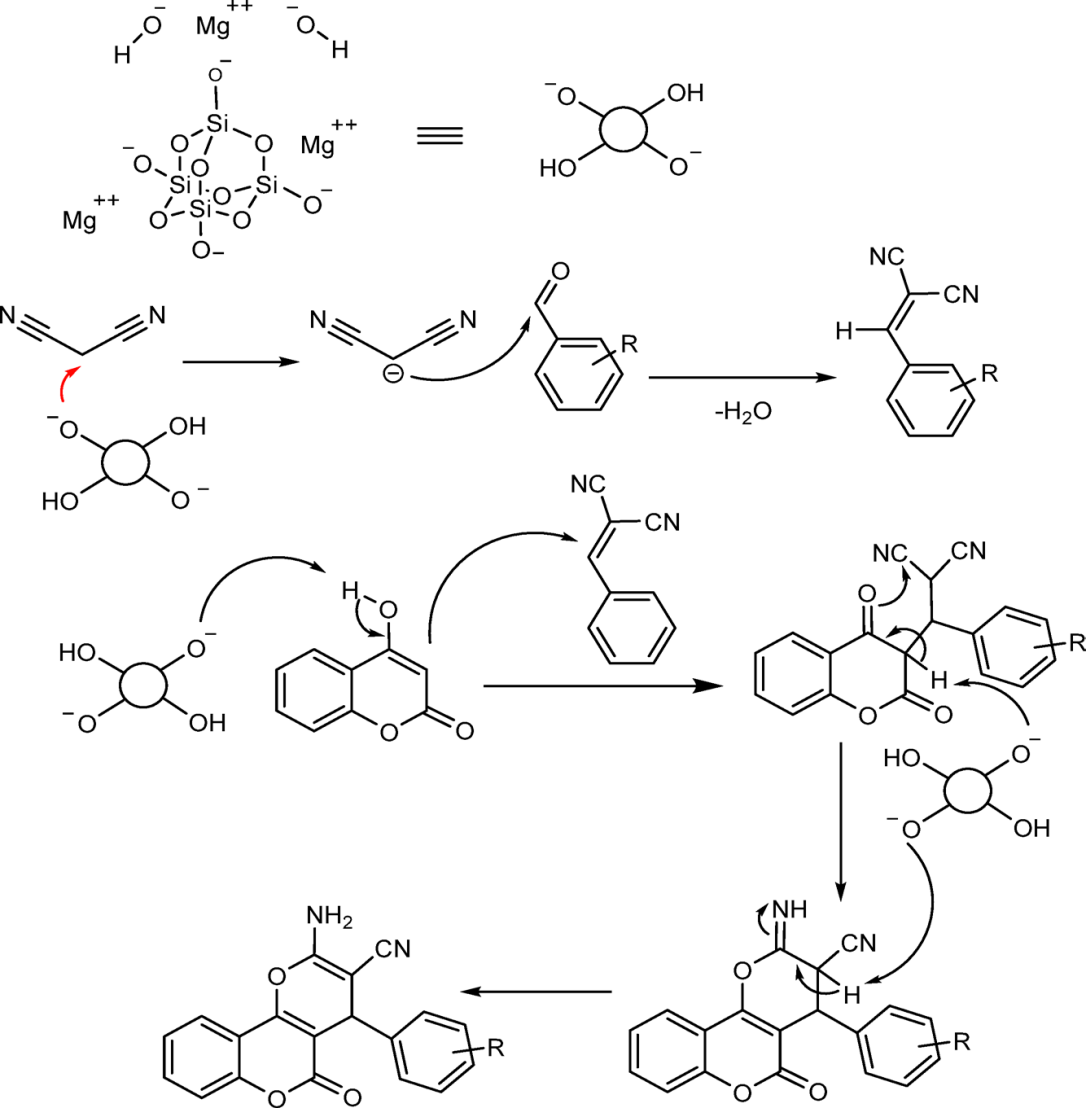
Proposed mechanism for the talc powder catalyzed synthesis of dihydropyranochromenes.

To evaluate the catalytic role of talc powder in the synthesis of dihydropyranochromanes, the reaction was conducted in ethanol under reflux conditions, both with and without the catalyst. The results showed a yield of only 10% for dihydropyranochromanes after 90 min without the catalyst, whereas a remarkable 95% yield was achieved in the presence of talc powder after the same duration. These findings underscore the necessity of a catalyst in the reaction. The proposed roles of talc powder as a catalyst in the reaction mechanism are illustrated in Fig. 6.

**Fig. 7:**
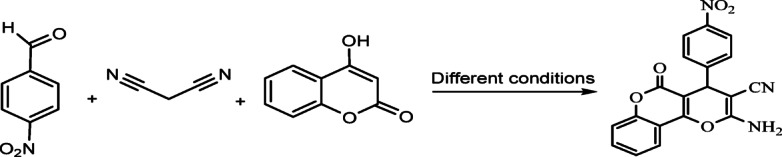
Synthesis of 2-Amino-4-(4-nitrophenyl)−5-oxo-4,5-dihydropyrano[3,2-*c*]chromene-3-carbonitrile under different conditions.

**Fig. 8:**
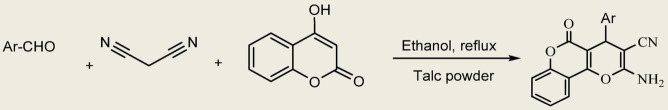
Synthesis of dihydropyranochromenes in the presence of Talc powder under reflux condition in ethanol.

The talc catalyst was employed under various conditions for the synthesis of the dihydropyranochromenes (Fig. 7). To optimize the reaction parameters, 4-nitrobenzaldehyde (1 mmol), 4-hydroxy coumarin (1 mmol), and malononitrile (1 mmol) were subjected to different experimental conditions, including varying amounts of catalyst and the usage of a mixer mill operating at frequencies of 10, 15, and 20 Hz at room temperature (Table 3). In addition, the reactions were conducted using a thermal stirrer with various solvents. The progress of the reaction was monitored using TLC in a solvent system of n-hexane and ethyl acetate (4:1). At the conclusion of the reaction, the catalyst was separated, and the product was worked up by adding water. Under the optimized reaction conditions (as detailed in Table 3, entry 11), the best results were achieved using ethanol as the solvent and 0.06 g of catalyst (Fig. 8; Table 4).

**Table 3:** Comparative performances of the Talc catalyst for the synthesis of dihydropyranochromene in different conditions.

Entry	Condition solvent/catalyst (g)	Condition	Time (min)	Yield (%)^a^
1	–/Talc (0.04)	Mixer Mil 10 Hz	20	Trace
2	–/Talc (0.04)	Mixer Mil 15 Hz	20	Trace
3	–/Talc (0.04)	Mixer Mil 20 Hz	20	Trace
4	–/Talc (0.06)	Mixer Mil 20 Hz	20	Trace
5	–/-	Reflux	90	Trace
6	H_2_O/-	Reflux	90	Trace
7	C_2_H_5_OH/-	Reflux	90	10
8	C_2_H_5_OH/Talc (0.01)	Reflux	90	43
9	C_2_H_5_OH/Talc (0.02)	Reflux	90	61
10	C_2_H_5_OH/Talc (0.04)	Reflux	90	78
11	C_2_H_5_OH/Talc (0.06)	Reflux	90	95
12	C_2_H_5_OH: H_2_O/Talc (0.04)	R.T.	120	57
13	C_2_H_5_OH/NaOH (0.04)	Reflux	20	50^b^
14	C_2_H_5_OH/K_2_CO_3_ (0.04)	Reflux	180	95^b^
15	C_2_H_5_OH/MgO (0.04)	Reflux	120	98^b^
16	C_2_H_5_OH/Hydrotalcite (0.04)	Reflux	150	95^b^

The ratio of 4-nitrobenzaldehyde: malononitrile:4-hydroxycoumarine is 1 mmol: 1.2 mmol: 1 mmol.

^a^Isolated Yield, ^b^Conversion yield.

**Table 4:** Synthesis of dihydropyranochromene derivatives in the presence of Talc powder.

Comp.	ArCHO	Product	Time (min)	Yield (%)^a^	A.E^b^ (%)	E.F^c^	M.*P*. (˚C)	M.*P*. (˚C) (rep.)^ref^.
1_b_	4-NO_2_-Ph		90	95	95	0.1	256–259	258–260^35^
2_b_	4-OH-Ph		120	87	94	0.2	266–268	265–267^36^
3_b_	3-NO_2_-Ph		120	89	95	0.18	260–263	261–263^36^
4_b_	2-NO_2_-Ph		90	92	95	0.14	241–243	242–245^35^
5_b_	2-Cl-Ph		90	88	95	0.19	262–264	264–266^35^
6_b_	4-MeO-Ph		90	87	95	0.2	247–250	246–248^36^
7_b_	2,6-Cl_2_-Ph		15	85	95.5	0.23	253–256	256–258^36^
8_b_	Ph		12	83	94.5	0.27	256–258	256–258^35^
9_b_	4-Cl-Ph		5	91	95	0.15	262–265	263–265^35^

^a^The ratio of aldehyde: malononitreile: 4-hydroxycoumarine: talc powder is 1 mmol:1.2 mmol:1 mmol:0.06 g.

^b^Isolated Yield.

^c^Atomic Economy.

^d^E-Factor.

**Fig. 9:**
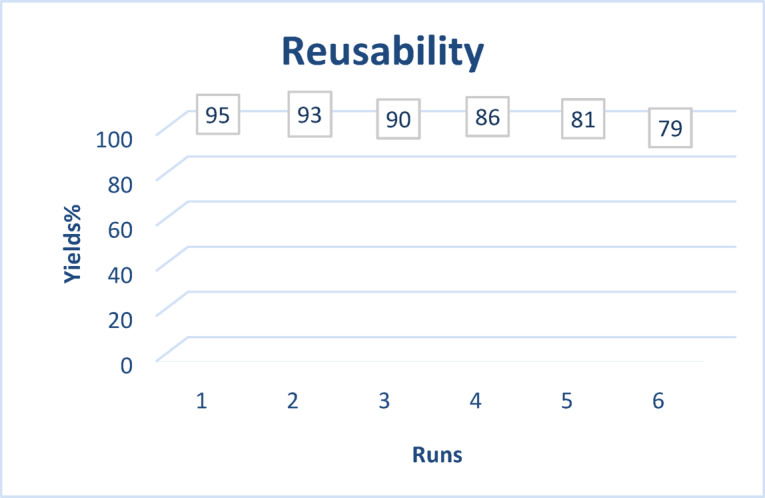
Reusability of Talc powder for the synthesis of dihydropyranochromene.

The reusability of catalyst in synthesis of dihydropyranochromene was studied in model reaction for six runs. After each run, the catalyst was separated from the reaction mixture by filtration and washed with ethanol, dried at room temperature and reused in next run (Fig. 9).

## Computational methods

In this article, we computed the stability of pyranopyrazoles and dihydropyranochromene derivatives using density functional theory (DFT). The simulations were carried out using the Becke-3-Lee–Yang–Parr (B3LYP) method, with a 6–311 G (d, p) basis set, in the Gaussian 09 software^37–43^. The HOMO-LUMO energy simulations were performed to evaluate the energetic behaviour of the compounds. The optimization and visualization of the charge distribution in the compounds were carried out with the GaussView 05 software.

The calculations of E_LUMO,_ E_HOMO_, band gap (E_g_=E_LUMO_-E_HOMO_) and the total energy for all dihydro pyranochromene derivatives and pyranopyrazoles are illustrated in Tables 5 and 6. The results of these tables indicate that the stability of compounds 7_a_ and 7_b_ is greater than that of the other reported compounds, which is consistent with the experimental data^44^.

**Table 5:** The calculations of E_LUMO,_ E_HOMO_, band gap (E_g_=E_LUMO_-E_HOMO_) and the total energy for Pyrano pyrazoles.

Comp.	E_tot_ (a.u)	E_HOMO_	E_LUMO_	E_g_=E_LUMO_-E_HOMO_
1_a_	− 1001.333758	− 0.2208	− 0.2052	0.015595
2_a_	− 872.1065607	− 0.2055	− 0.1899	0.015548
3_a_	− 1256.4913548	− 0.2129	− 0.1971	0.015746
4_a_	− 911.3989460	− 0.2114	− 0.1958	0.015580
5_a_	− 836.2259554	− 0.2051	− 0.1898	0.015262
6_a_	− 747.2975442	− 0.2070	− 0.1963	0.010690
7_a_	− 1716.0609398	− 0.2050	− 0.1890	0.015970
8_a_	− 786.5830975	− 0.2070	− 0.1919	0.015010
9_a_	− 895.3630129	− 0.2056	− 0.1900	0.015555

**Table 6:** The calculations of E_LUMO,_ E_HOMO_, band gap (E_g_=E_LUMO_-E_HOMO_) and the total energy for Dihydropyrano chromenes.

Comp.	E_tot_ (a.u)	E_HOMO_	E_LUMO_	E_g_=E_LUMO_-E_HOMO_
1_b_	− 1320.1750075	− 0.2081	− 0.1656	0.04246
2_b_	− 1140.0236481	− 0.2055	− 0.1660	0.03950
3_b_	− 1269.2527338	− 0.2129	− 0.1726	0.04022
4_b_	− 1269.2635701	− 0.2014	− 0.1596	0.04175
5_b_	− 1524.39483219	− 0.2051	− 0.1612	0.04388
6_b_	− 1179.3177055	− 0.2070	− 0.1669	0.04010
7_b_	− 1948.61395248	− 0.2049	− 0.1110	0.09387
8_b_	− 1064.8363025	− 0.2070	− 0.1677	0.03930
9_b_	− 1524.4098279	− 0.2056	− 0.1556	0.04996

**Fig. 10:**
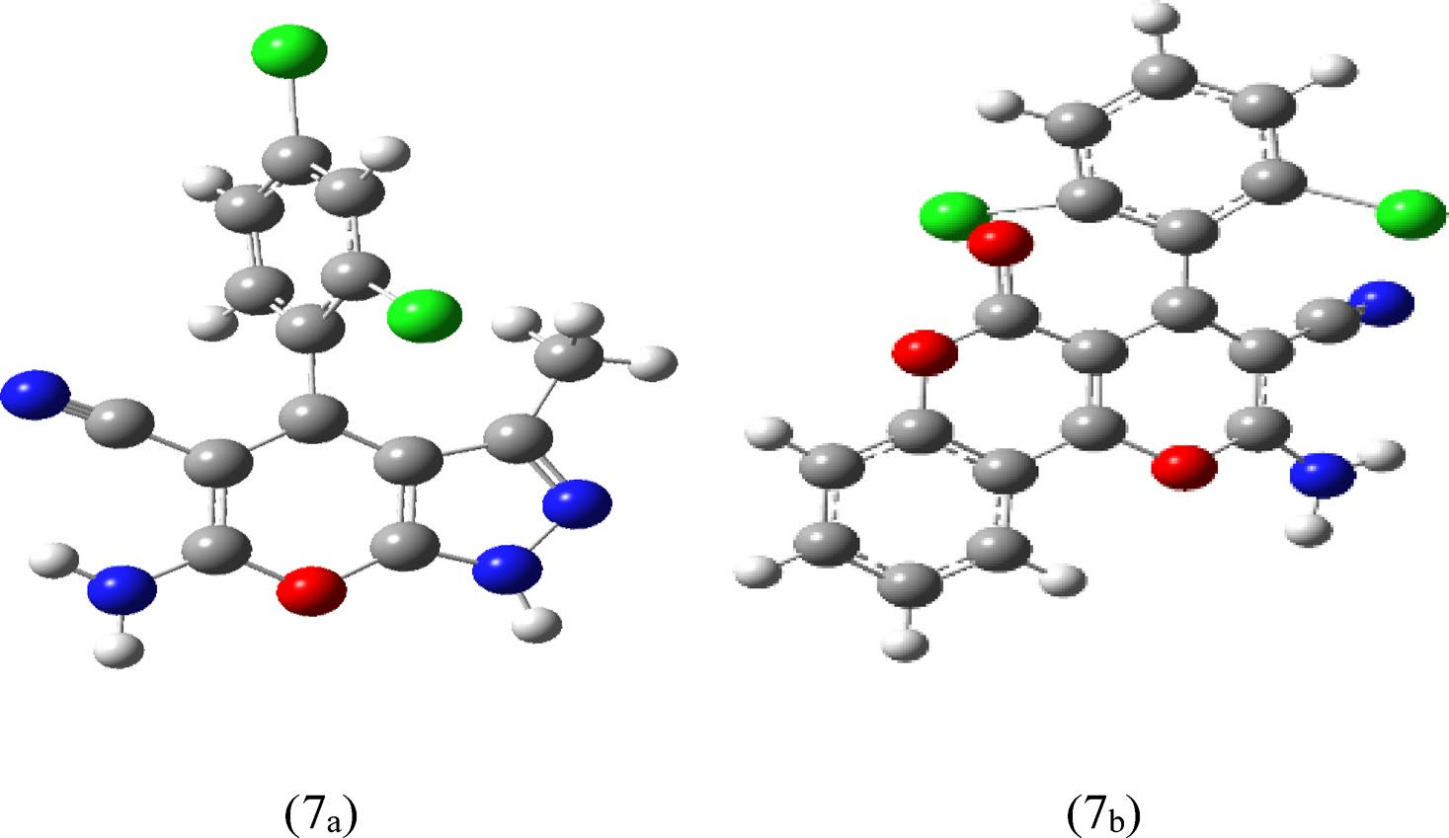
The relaxed structures for compound 7_a_ and 7_b_.

**Fig. 11:**
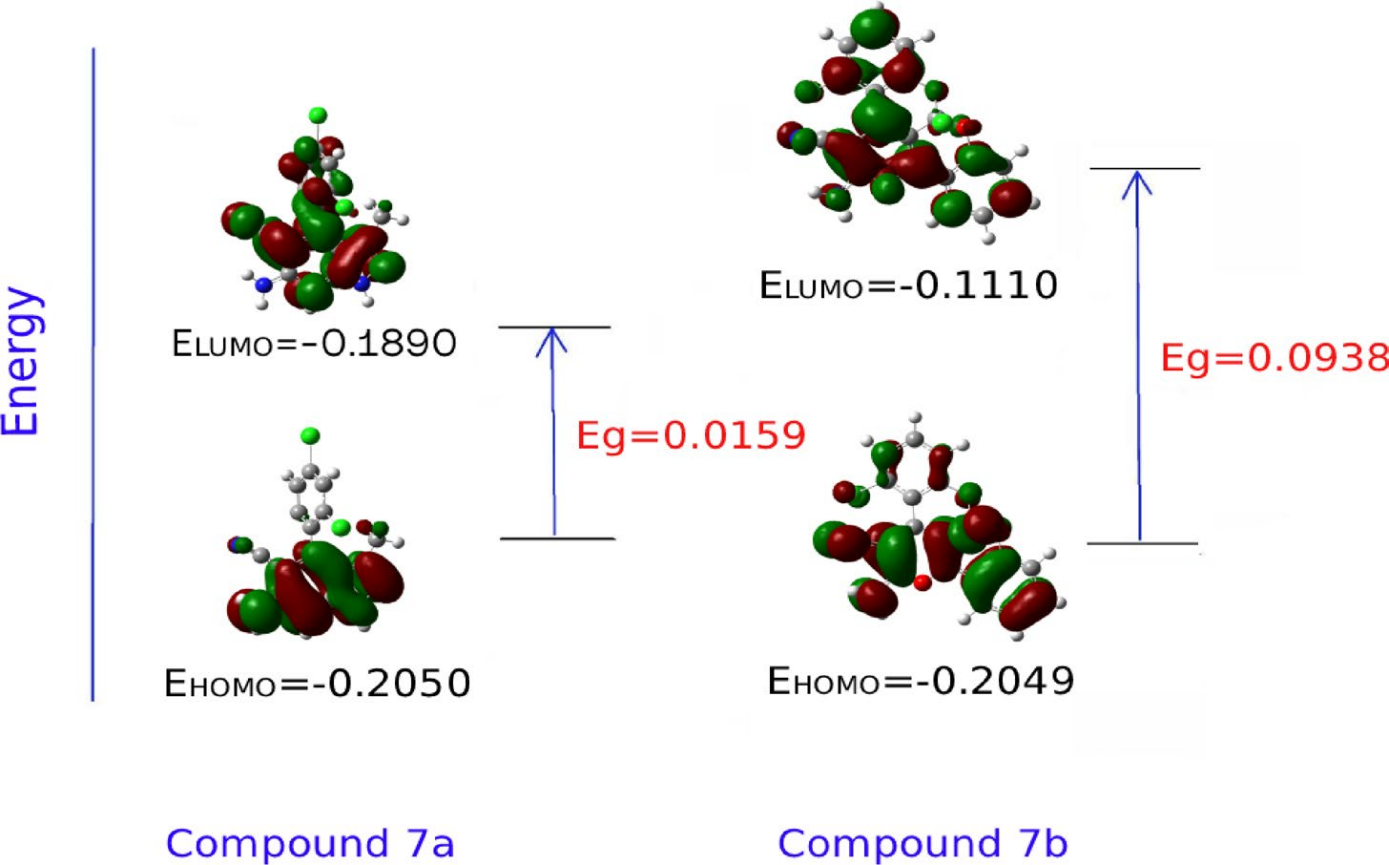
Frontier molecular orbital diagram for 7_a_ and 7_b_ compounds.

Additionally, Figs. 10 and 11 showcase the optimized structures and the frontier molecular orbital diagram related to compounds 7_a_ and 7_b_, respectively.

## Experimental

### Materials and methods

All chemicals were purchased from Merck and Sigma-Aldrich without further purification. We have used the exact same talc material from our earlier publication^31^ without any modification. All yields refer to isolated products, which were characterized by spectral data. FT-IR spectra were run on a Bruker Equinox 55 spectrometer. The nuclear magnetic resonance (NMR) spectra were recorded in CDCl_3_ or DMSO-d_6_ on a Bruker Avance NMR 400 MHz. The melting points were determined on a Buchi B-540 apparatus. A mixer mill model Retsch MM 400, which consisted of two stainless steel vials, was used.

### Synthesis of pyranopyrazole in the presence of talc powder

In a stainless-steel mixer mill vessel, a mixture of ethylacetoacetate (1 mmol), hydrazine hydrate (1.5 mmol), aldehydes (1 mmol), malononitrile (1 mmol), and talc powder (0.04 g) was milled at 20 Hz. The reaction progress was monitored by TLC (n-hexane: ethyl acetate [4:1]). After completion of the reaction, hot ethanol was added, and the products were easily separated from the catalyst and purified using recrystallization in ethanol.

### Synthesis of dihydropyranochromene in the presence of talc powder

For synthesis of the dihydropyranochromene derivatives after determining the optimal conditions, a mixture of malononitrile (1 mmol), 4-hydroxycoumarin (1 mmol), benzaldehyde derivative (1 mmol), and talc (0.06 g) in ethanol (10 mL) was refluxed for an appropriate time. After completion of the reaction (monitored by TLC), the mixture was cooled to room temperature, and the catalyst was filtered. Then, separation was carried out by adding water to obtain the desired dihydropyranochromene derivatives.

### Hot filtration for synthesis of dihydropyranochromene

A mixture of malononitrile (1 mmol), 4-hydroxycoumarin (1 mmol), 4-nitrobenzaldehyde derivative (1 mmol), and talc (0.06 g) in ethanol (10 mL) was refluxed for 45 min. The progress of reaction was monitored by TLC. The conversion yield was 60–65%. In this step, the catalyst was removed from reaction mixture by filtration. The filtrate was refluxed for another 45 min. The conversion yield was 65–70%. This evidence shows that the catalyst is heterogeneous ones with no any leaching in reaction m.

edium.

## Conclusions

This paper has demonstrated the versatility of talc powder as a catalyst in multicomponent reactions for the synthesis of pyranopyrazoles and dihydropyranochromanes. The use of talc powder in both solution phase and solvent-free mixer milling conditions has provided efficient, environmentally friendly, and economically viable routes for the synthesis of these important heterocyclic compounds. This suggests that talc powder, as a natural catalyst, can enhance organic synthesis and offer a sustainable and environmentally friendly catalyst for modern organic chemistry. The DFT simulations indicate that compound 7 is more stable for both dihydropyranochromenes and pyranopyrazoles than the other reported compounds, aligning with the experimental data.

## Supplementary Information

Below is the link to the electronic supplementary material.

Supplementary Material 1

## Data Availability

All data generated or analyzed during this study are included in supplementary.
