# Supplementary material for: Natural talc: a basic, cost-effective, and available catalyst for the one-pot synthesis of dihydropyranochromenes and pyranopyrazoles, along with related DFT calculations
Source: Sci Rep. 2025 Dec 14;16:2464. doi: 10.1038/s41598-025-32187-4 (PMC12820251; doi:10.1038/s41598-025-32187-4)
Supplement: Supplementary file 1 — Supplementary Material 1 [file 41598_2025_32187_MOESM1_ESM.docx]

Natural talc: A basic, cost-effective, and available catalyst for the one-pot synthesis of dihydropyranochromenes and pyranopyrazoles, along with related DFT calculations

Abolfazl Dehghanizadeh, Bi Bi Fatemeh Mirjalili,* Hadi Basharnavaz*

Department of Chemistry, College of Science, Yazd University, Yazd, P.O. Box 89195-741, Iran

*E-mail: fmirjalili@yazd.ac.ir*

***6-Amino-3-methyl-4-(4-nitrophenyl)-1,4-dihydropyrano[2,3-c]pyrazole-5-carbonitrile***: White solid. M. p. = 243-245 °C. FT- IR (ATR)/ ῡ(cm^-1^):3219, 3103, 2195, 1646, 1593, 1514, 1403, 1351, 1163, 1108, 810, 746.; ^1^H-NMR (400 MHz, Acetone-d_6_)/ δ ppm: 2 (s, 3H), 4.88 (s, 1H), 6.30 ( s, 2H), 7.55 (d, *J* = 8 Hz, 2H), 8.23 (d, *J* = 8 Hz, 2H), 11.43 (s, 1H). ^13^CNMR (100 MHz, DMSO-d_6_)/δ ppm: 161.62, 155.15, 152.59, 146.85, 136.36, 132.19, 129.32, 124.38, 120.98, 97.04, 56.37, 36.36, 10.22.

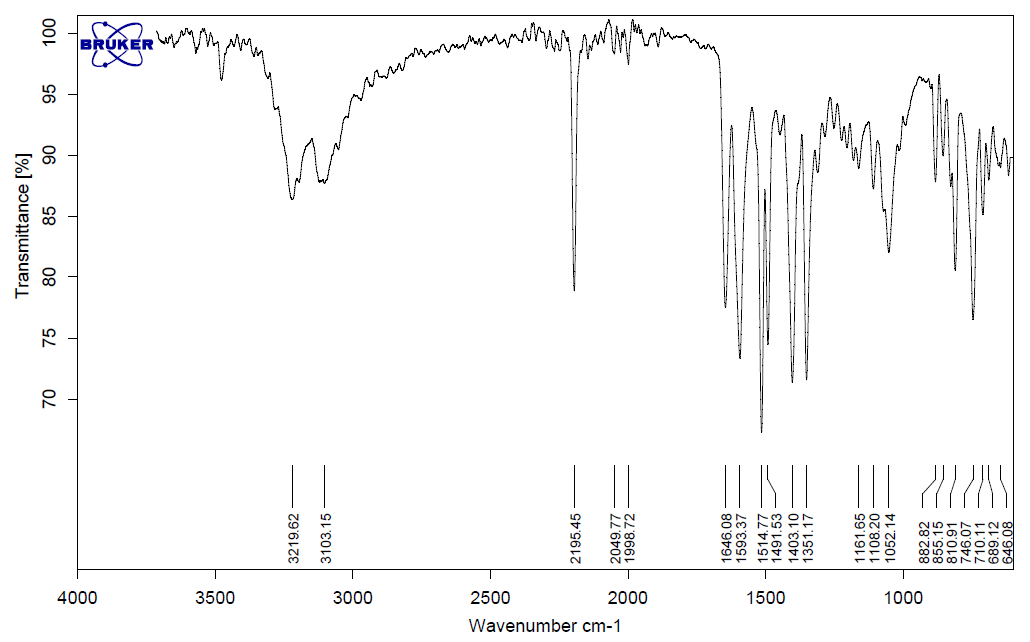


The FT-IR of 6-Amino-3-methyl-4-(4-nitrophenyl)-1,4-dihydropyrano[2,3-*c*]pyrazole-5-carbonitrile


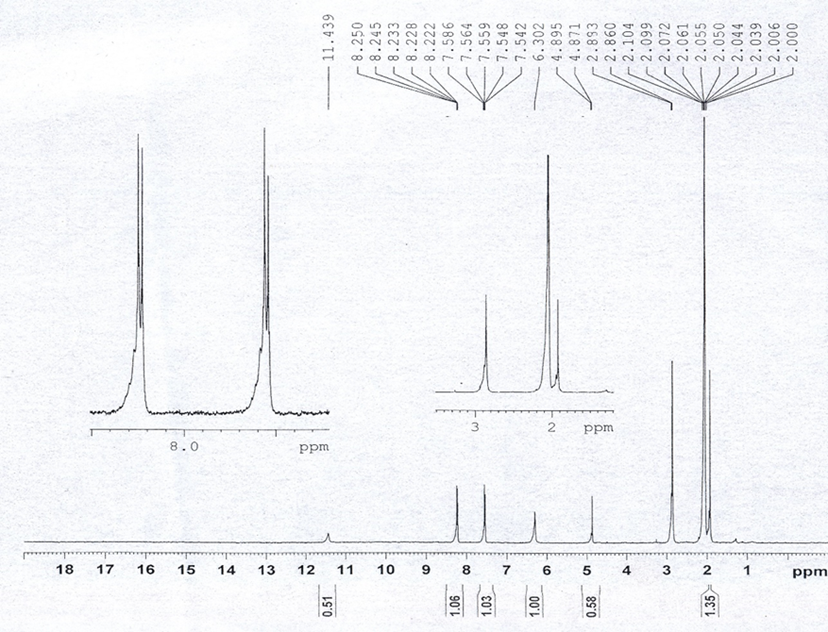


The ^1^H NMR (400 MHz) spectrum of 6-Amino-3-methyl-4-(4-nitrophenyl)-1,4-dihydropyrano[2,3-*c*]pyrazole-5-carbonitrile


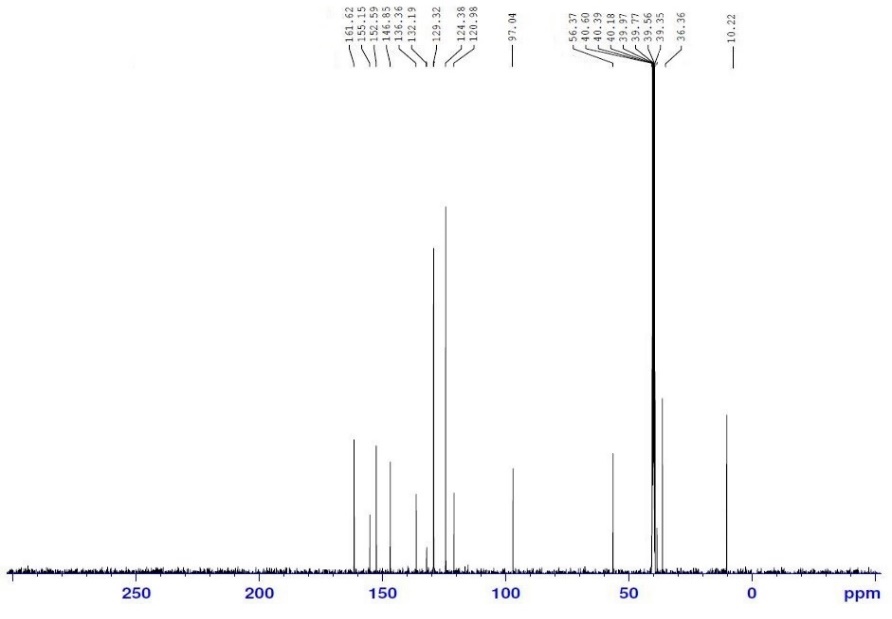


The ^13^C NMR (100 MHz) spectrum of 6-Amino-3-methyl-4-(4-nitrophenyl)-1,4-dihydropyrano[2,3-*c*]pyrazole-5-carbonitrile

***6-Amino-4-(4-hydroxyphenyl)-3-methyl-1,4-dihydropyrano[2,3-c]pyrazole-5-carbonitrile (Table 2, entry 8):*** Cream solid. M.p. 221-224 °C. FT- IR (ATR)/ ῡ(cm^-1^): 3367, 3134, 2175, 1646, 1596, 1512, 1491, 1406, 1190, 1044, 809. ^1^H NMR (400 MHz, Acetone-d_6_)/ δ(ppm): 1.74 (s, 3H), 4.44 (s, 1H), 6.65 (dd, *J*=7.5 Hz, *J*=3.7 Hz, 2H), 6.76 (brs, 2H), 6.91 (dd, *J*=7.5 Hz, *J*=3.7 Hz, 2H), 9.27 (s, 1H), 12.02 (s, 1H).; ^13^C NMR (100 MHz, DMSO-d_6_)/δ ppm: 161.10, 156.49, 155.22, 135.98, 135.24, 128.92, 121.40, 115.58, 98.54, 58.21, 35.95, 10.24.

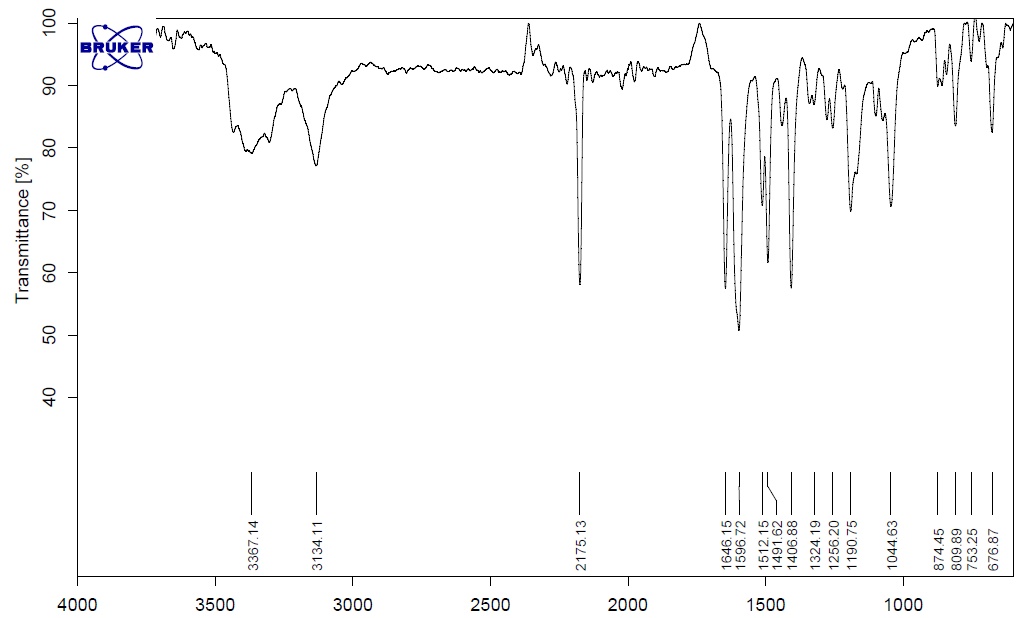


The FT-IR of 6-Amino-4-(4-hydroxyphenyl)-3-methyl-1,4-dihydropyrano[2,3-*c*]pyrazole-5-carbonitrile


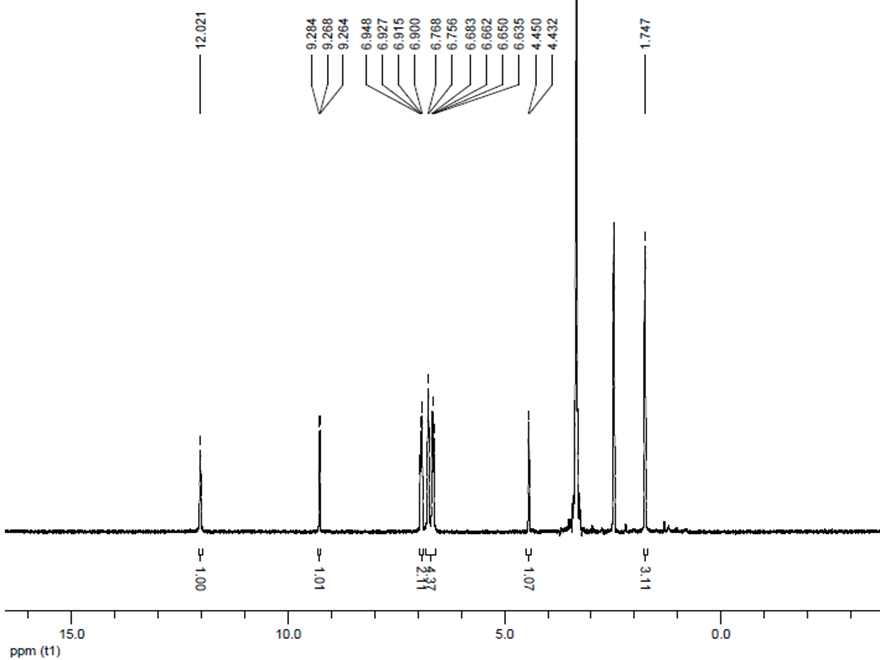


The ^1^H NMR (400 MHz) spectrum of 6-Amino-4-(4-hydroxyphenyl)-3-methyl-1,4-dihydropyrano[2,3-*c*]pyrazole-5-carbonitrile


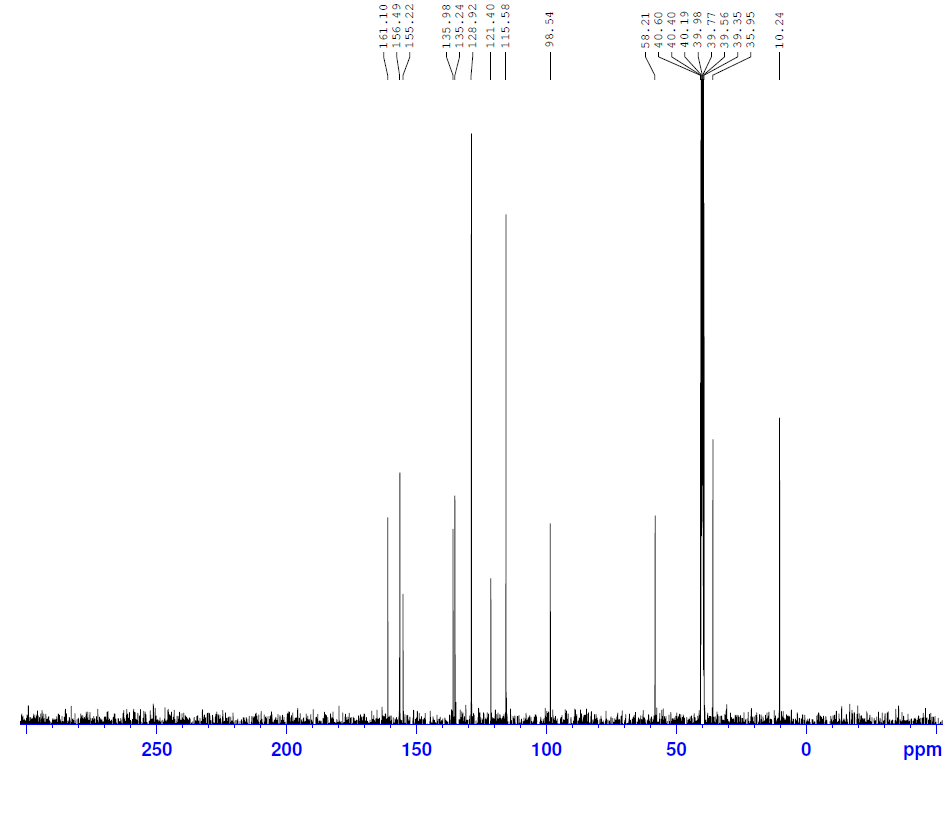


The ^13^C NMR (100 MHz) spectrum of 6-Amino-4-(4-hydroxyphenyl)-3-methyl-1,4-dihydropyrano[2,3-*c*]pyrazole-5-carbonitrile

***6-Amino-4-(4-chlorophenyl)-3-methyl-1,4-dihydropyrano[2,3-c]pyrazole-5-carbonitrile:*** White solid. M.p. 236-238 °C. FT- IR (ATR) /ῡ (cm^-1)^: 3124, 2192, 1640, 1594, 1489, 1054, 798. ^1^HNMR (400 MHz, DMSO-d_6_)/δ (ppm): 1.81 (s, 3H), 4.65 (s, 1H), 6.96 (s, 2H), 7.21 (d, *J* = 8 Hz, 2H), 7.39 (d, *J* = 8 Hz, 2H), 12.16 (s, 1H).; ^13^C NMR (100 MHz, DMSO-d_6_)/δ ppm: 161.38, 155.18, 143.97, 136.15, 131.71, 130.51, 129.85, 128.94, 121.15, 97.67, 57.19, 36.03, 10.22.

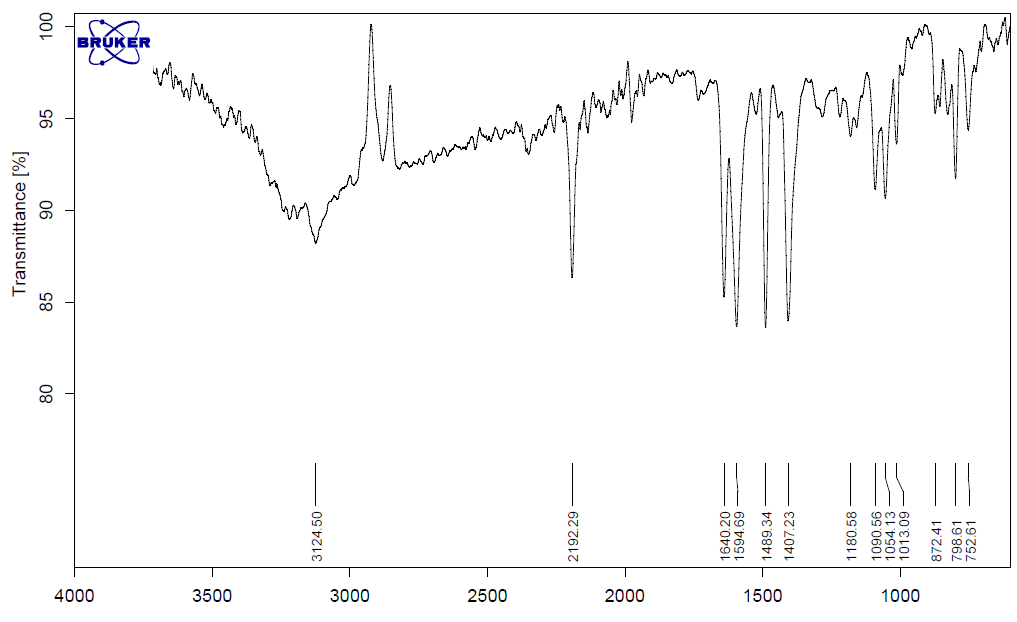


The FT-IR of 6-Amino-4-(4-chlorophenyl)-3-methyl-1,4-dihydropyrano[2,3-*c*]pyrazole-5-carbonitrile


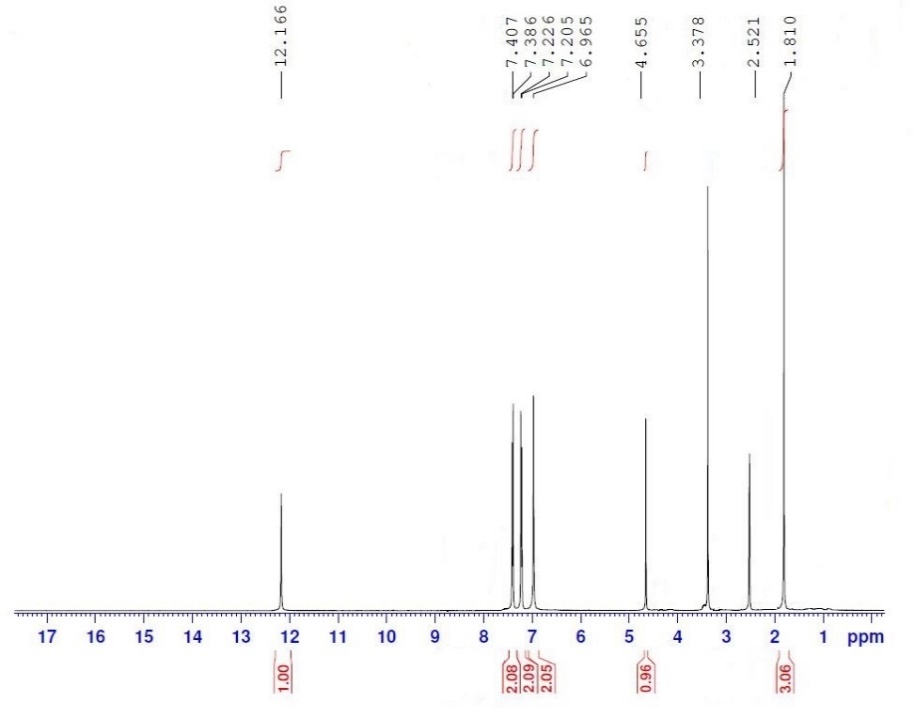


The ^1^H NMR (400 MHz) spectrum of 6-Amino-4-(4-chlorophenyl)-3-methyl-1,4-dihydropyrano[2,3-*c*]pyrazole-5-carbonitrile


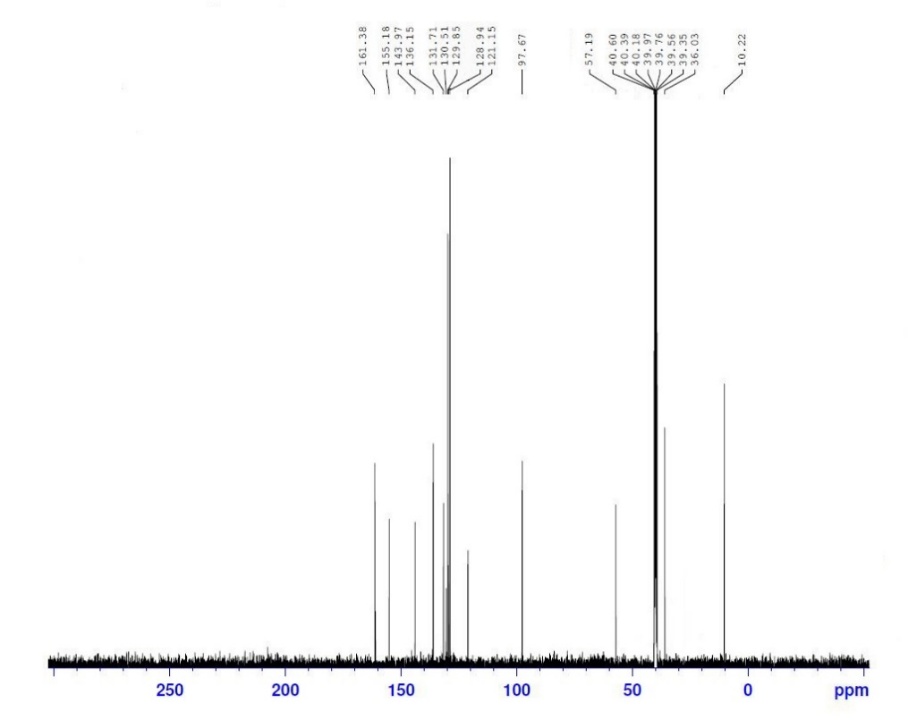


The ^13^C NMR (100 MHz) spectrum of 6-Amino-4-(4-chlorophenyl)-3-methyl-1,4-dihydropyrano[2,3-*c*]pyrazole-5-carbonitrile

***6-Amino-4-(2-methoxyphenyl)-3-methyl-1,4-dihydropyrano[2,3-c]pyrazole-5-carbonitrile.*** Yellow solid. M.p. 220-222 °C. FT- IR (ATR)/ ῡ(cm^-1^ ): 3374, 3310, 3154, 2193, 1597, 1486, 1241, 1159, 1104, 1026, 762.; ^1^HNMR (400 MHz, DMSO-d_6_)/ δ ppm: 1.88 (s, 3H), 3.87 (s, 3H), 5.60 (s, 1H), 6.89 (s, 2H), 6.99 (t, *J* = 7.2 Hz, 1H), 7.06-7.10 (m, 2H), 7.27-7.31 (m, 1H), 12.09 (s, 1H).; ^13^C NMR (100 MHz, DMSO-d_6_)/δ ppm: 161.44, 156.30, 155.04, 134.99, 132.07, 128.58, 127.87, 120.86, 120.78, 111.25, 97.79, 56.30, 55.53, 29.09, 9.47.


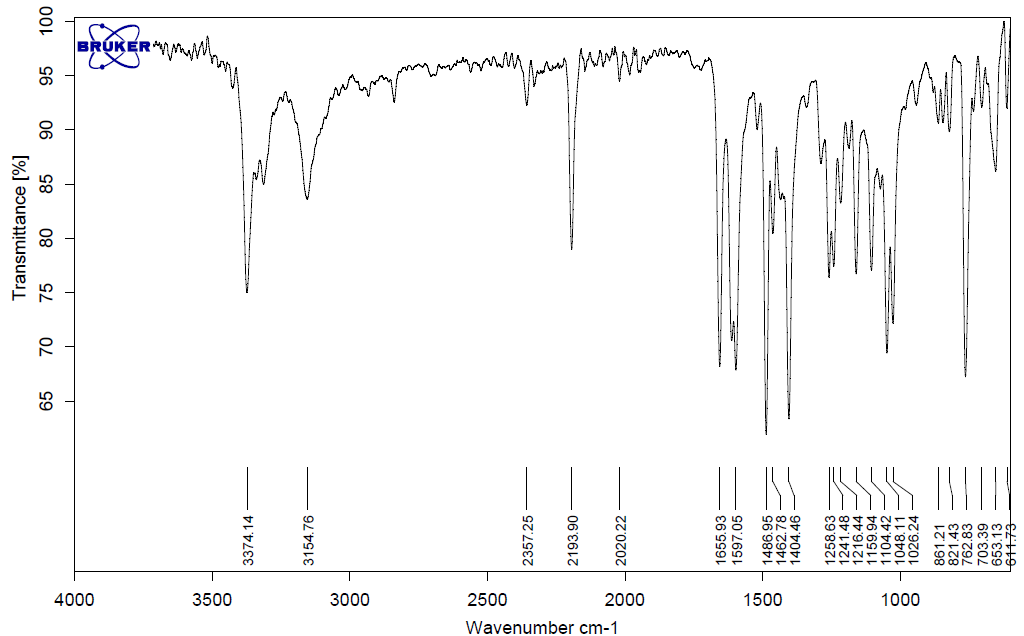


The FT-IR of 6-Amino-4-(2-methoxyphenyl)-3-methyl-1,4-dihydropyrano[2,3-*c*]pyrazole-5-carbonitrile


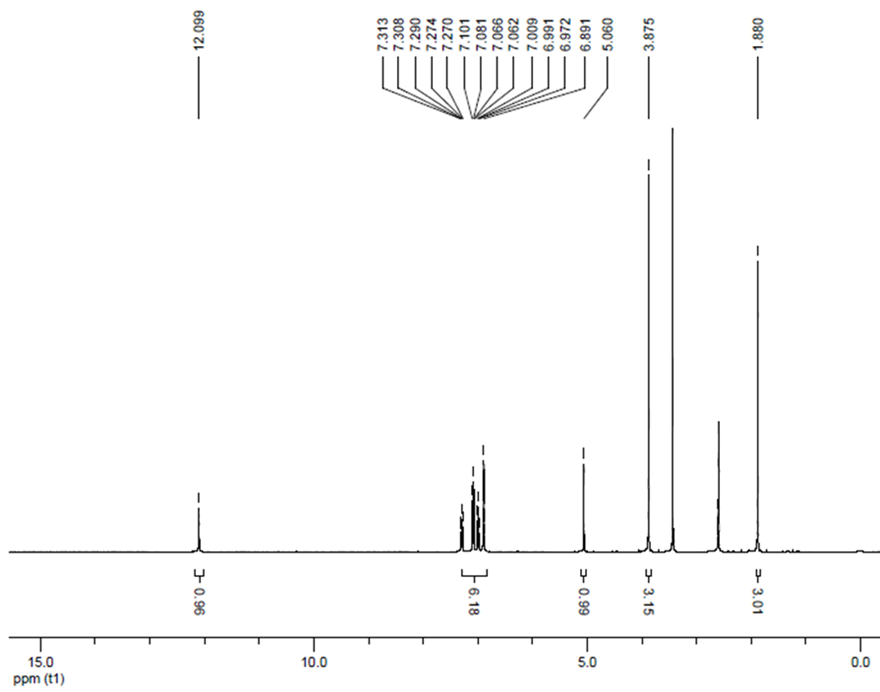


The ^1^H NMR (400 MHz) spectrum of 6-Amino-4-(2-methoxyphenyl)-3-methyl-1,4-dihydropyrano[2,3-*c*]pyrazole-5-carbonitrile


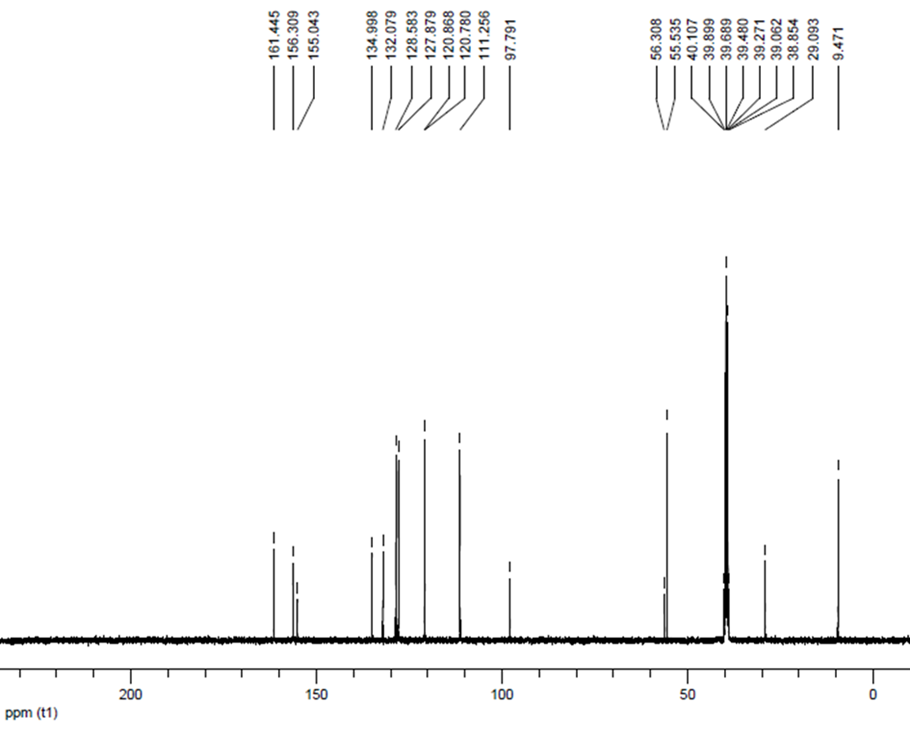


The ^13^C NMR (100 MHz) spectrum of 6-Amino-4-(2-methoxyphenyl)-3-methyl-1,4-dihydropyrano[2,3-*c*]pyrazole-5-carbonitrile

***6-Amino-3-methyl-4-(p-tolyl)-1,4-dihydropyrano[2,3-c]pyrazole-5-carbonitrile:*** Cream solid. M.p. 171-173 °C. FT- IR (ATR)/ ῡ(cm^-1^): 3360, 3171, 2182, 1649, 1598, 1490, 1401, 1046, 871.; ^1^H-NMR(400 MHz, DMSO-d_6_)/ δ ppm: 1.84 (s, 3H), 2.33 (s, 3H), 4.60 (s, 1H), 6.89 (s, 2H), 7.10 (d, *J* = 8 Hz, 2H), 7.17 (d, *J* = 8 Hz, 2H), 12.13 (s, 1H).


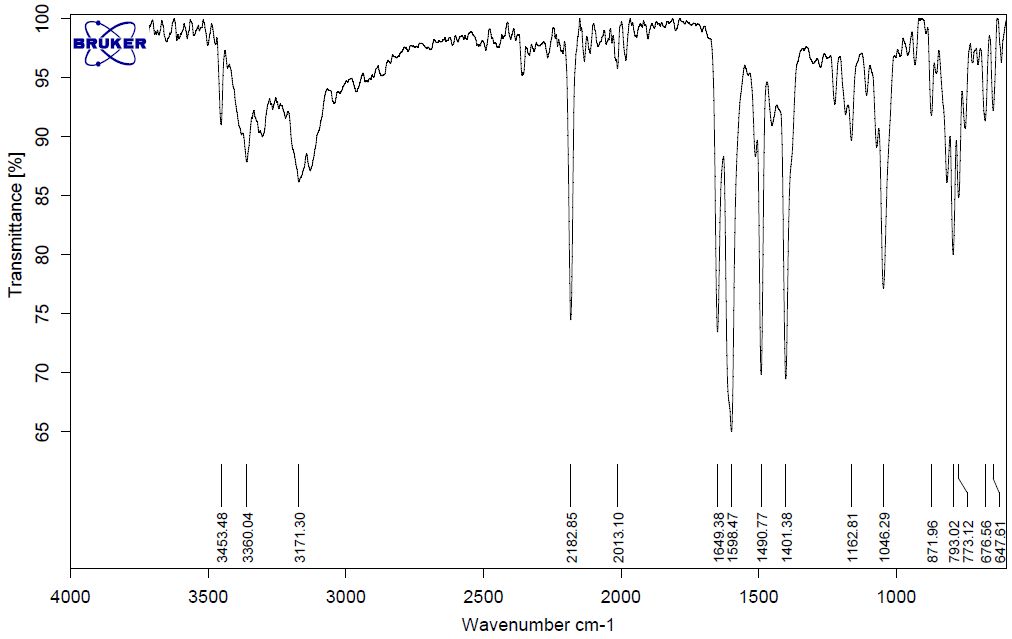


The FT-IR of 6-Amino-3-methyl-4-(p-tolyl)-1,4-dihydropyrano[2,3-c]pyrazole-5-carbonitrile


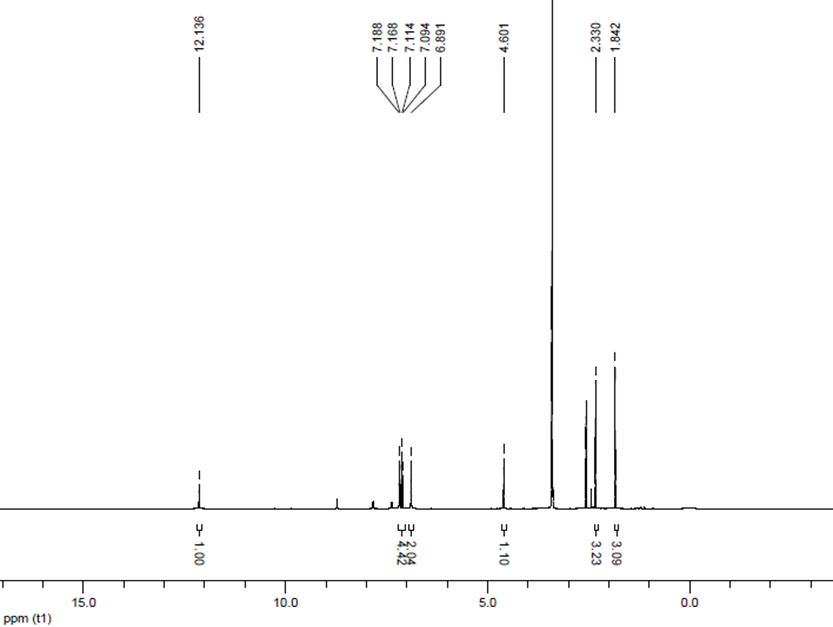


The ^1^H NMR (400 MHz) spectrum of 6-Amino-3-methyl-4-(p-tolyl)-1,4-dihydropyrano[2,3-c]pyrazole-5-carbonitrile

***6-Amino-4-(3,4-dihydroxyphenyl)-3-methyl-1,4-dihydropyrano[2,3-c]pyrazole-5-carbonitrile.*** Brown solid. M.p. 222-225 °C. FT- IR (ATR)/ ῡ(cm^-1^): 3458, 3250, 3125, 2180, 1628, 1595, 1492, 1339, 1265, 1051, 755.; ^1^H NMR (400 MHz, DMSO-d_6_)/ δ ppm: 1.78 (s, 3H), 4.35 (s, 1H), 6.42-6.45 (m, 2H), 6.61 (dd, *J* = 7.5 Hz, *J* = 1.5 Hz, 1H), 6.75 (s, 2H), 8.70 (s, 1H), 8.82 (s, 1H), 12.02 (s, 1H). ^13^C NMR (100 MHz, DMSO-d_6_)/δ ppm: 161.06, 155.21, 145.67, 144.52, 136, 121.44, 118.75, 115.61, 115.04, 98.62, 58.31, 36.14, 10.26

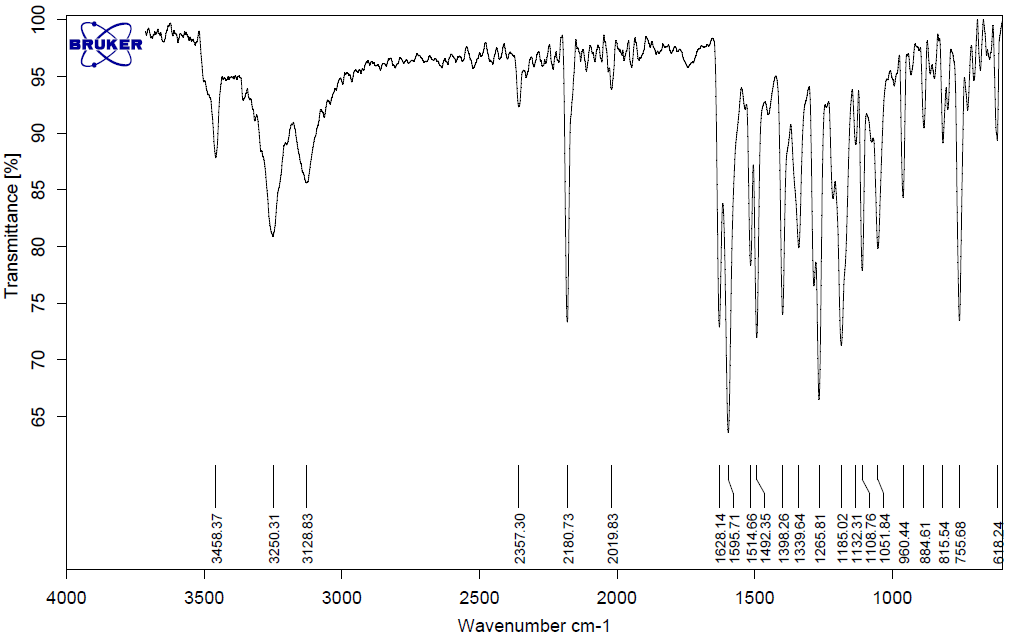


The FT-IR of 6-Amino-4-(3,4-dihydroxyphenyl)-3-methyl-1,4-dihydropyrano[2,3-c]pyrazole-5-carbonitrile


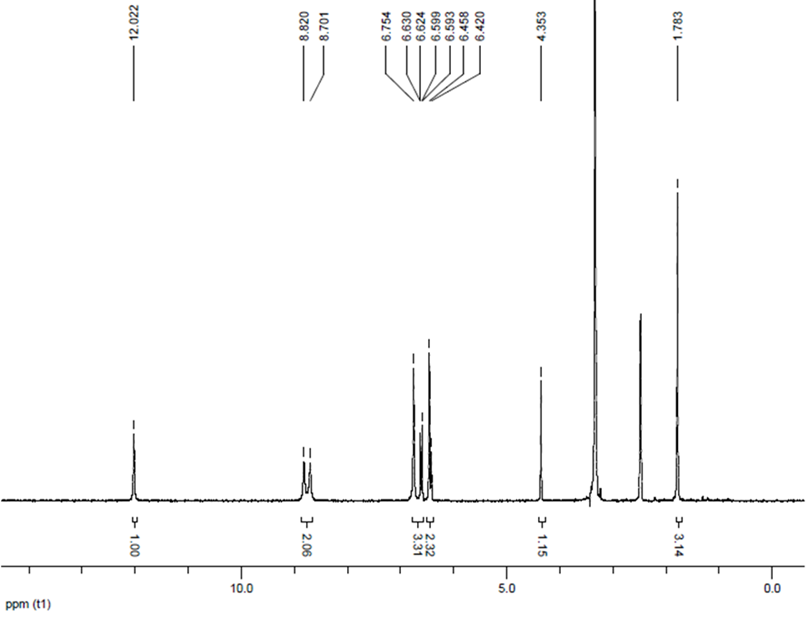


The ^1^H NMR (400 MHz) spectrum of 6-Amino-4-(3,4-dihydroxyphenyl)-3-methyl-1,4-dihydropyrano[2,3-c]pyrazole-5-carbonitrile


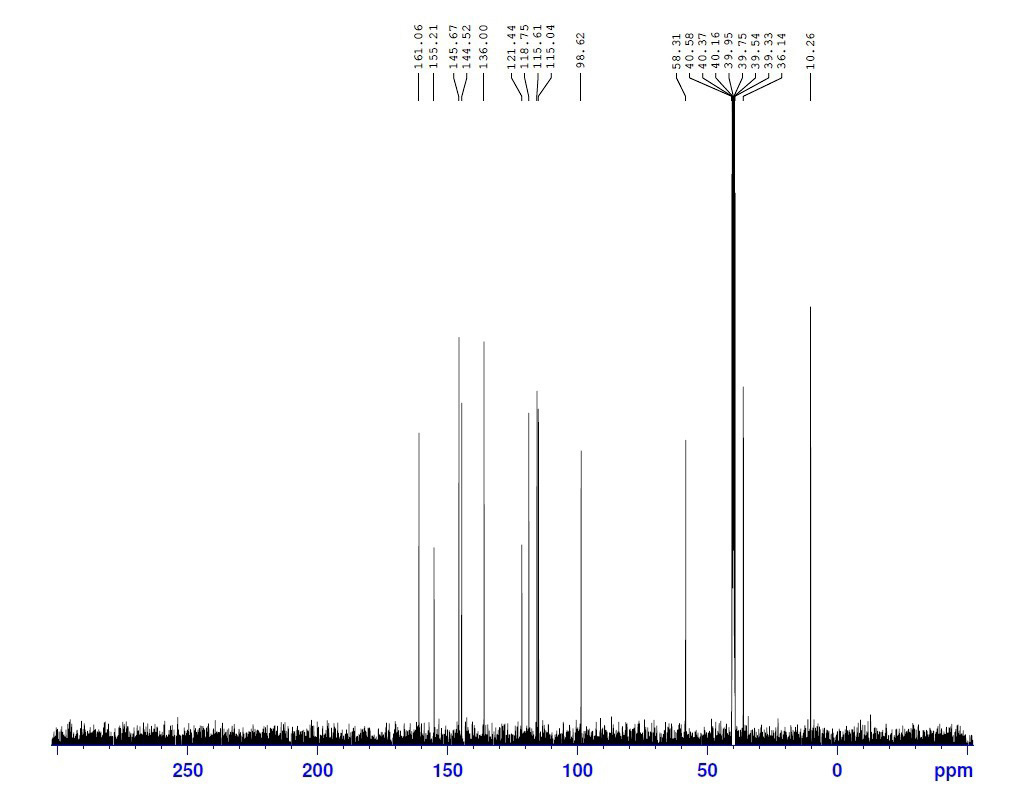


The ^13^C NMR (100 MHz) spectrum of 6-Amino-4-(3,4-dihydroxyphenyl)-3-methyl-1,4-dihydropyrano[2,3-c]pyrazole-5-carbonitrile

***6-Amino-4-(2,4-dichlorophenyl)-3-methyl-1,4-dihydropyrano[2,3-c]pyrazole-5-carbonitrile:*** Pale yellow solid. M.p. 223-226°C. FT- IR (ATR)/ ῡ(cm^-1^): 3394, 3295, 3120, 2177, 1649, 1591, 1494, 1409, 1098, 1050, 861, 750.; ^1^H NMR (400 MHz, DMSO-d_6_)/δ ppm: 1.85 (s, 3H), 5.13 (s, 1H), 7.07 (s, 2H), 7.29 (d, J = 8 Hz, 1H), 7.47 (dd, J = 8.4 Hz, J = 2 Hz, 1H), 7.65 (d, J = 2.4 Hz, 1H), 12.23 (s, 1H).; ^13^C NMR (100 MHz, DMSO-d_6_)/δ ppm: 161.30, 154.88, 140.07, 135.44, 132.81, 132.10, 128.83, 128.02, 120.25, 96.32, 55.21, 33.07, 9.53.


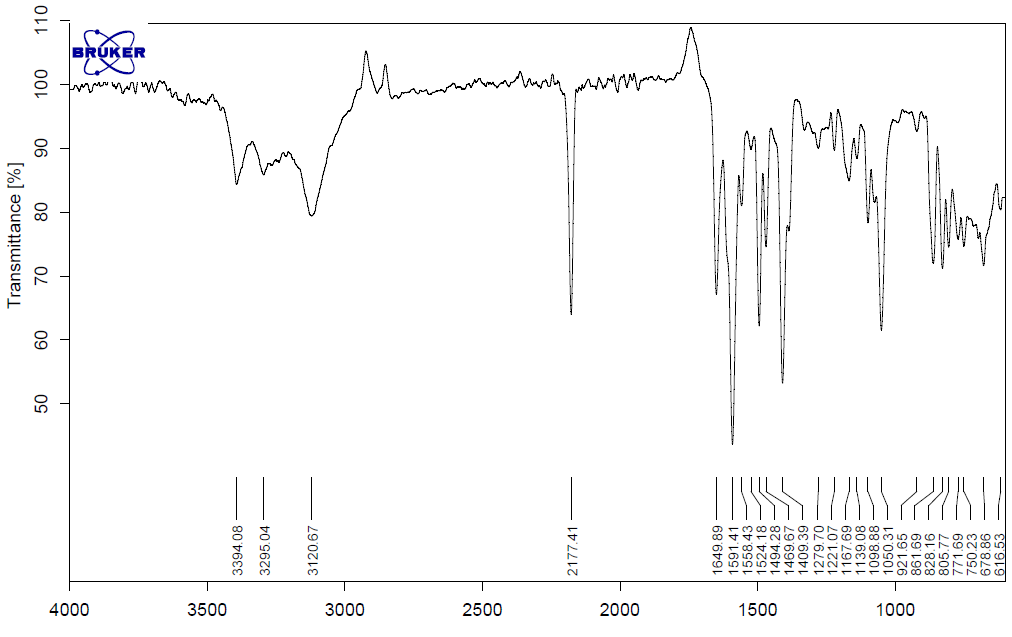


The FT-IR of 6-Amino-4-(2,4-dichlorophenyl)-3-methyl-1,4-dihydropyrano[2,3-*c*]pyrazole-5-carbonitrile


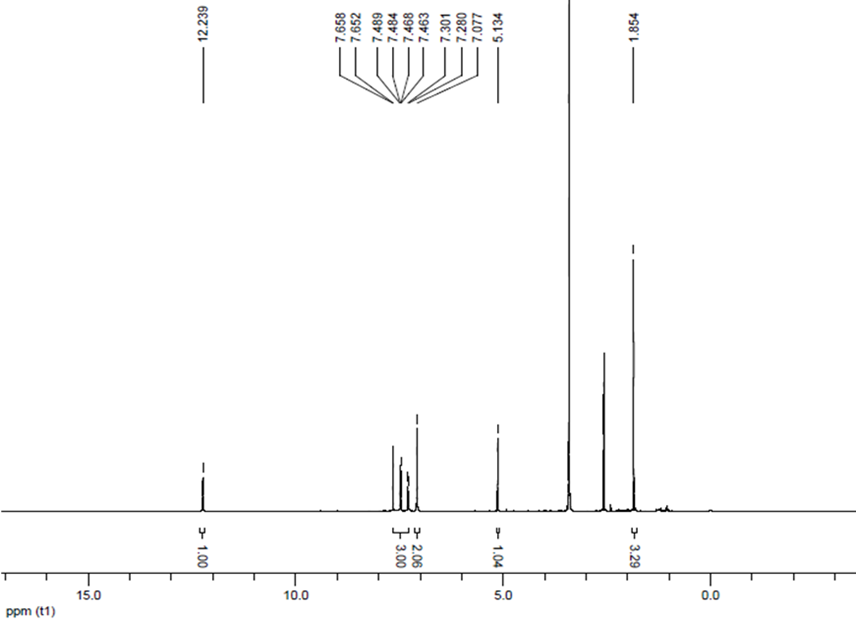


The ^1^H NMR (400 MHz) spectrum of 6-Amino-4-(2,4-dichlorophenyl)-3-methyl-1,4-dihydropyrano[2,3-*c*]pyrazole-5-carbonitrile


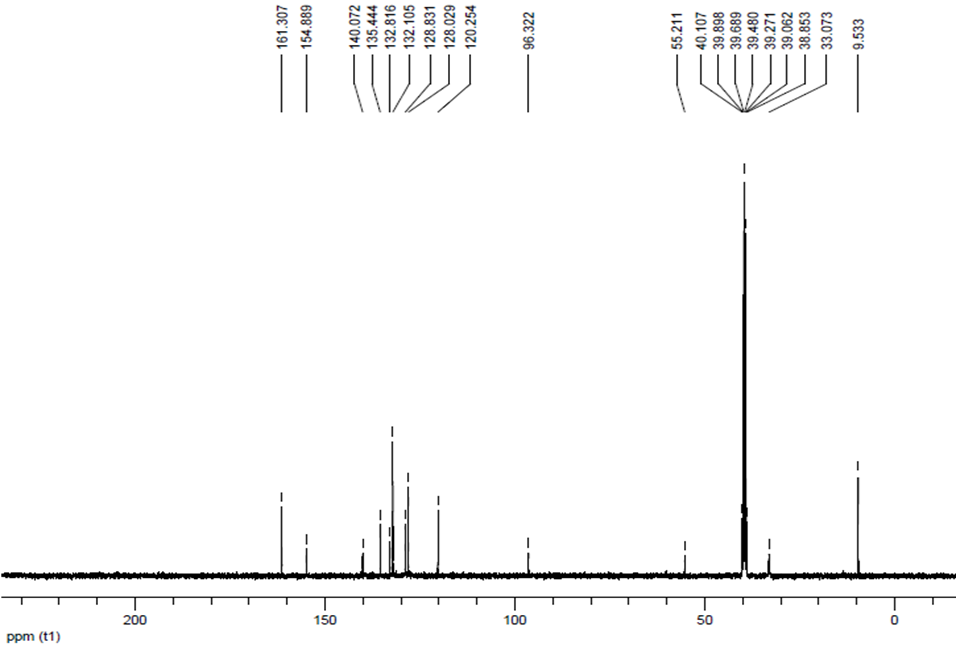


The ^13^C NMR (100 MHz) spectrum of 6-Amino-4-(2,4-dichlorophenyl)-3-methyl-1,4-dihydropyrano[2,3-*c*]pyrazole-5-carbonitrile

***6-Amino-4-(4-hydroxy-3-methoxyphenyl)-3-methyl-1,4-dihydropyrano[2,3-c]pyrazole-5-carbonitrile:*** Orange solid. M.p. 233-235 °C. FT-IR (ATR)/ ῡ(cm^-1^): 3488, 3406, 3325,3271, 3217, 2194, 1654, 1601, 1510, 1262, 1027, 743. ^1^H-NMR (400 MHz, DMSO-d_6_)/ δ ppm: 1.83 (s, 3H), 3.73 (s, 3H), 4.51 (s, 1H), 6.57 (s, 1H), 6.73-6.71 (d, *J* = 8 Hz, 2H), 6.83-6.88 (m, 2H), 9.73(s, 1H), 12.07 (s, 1H). ; ^13^C NMR (100 MHz, DMSO-d_6_)/δ ppm: 161.17, 155.19, 147.79, 145.70, 136.05, 123.99, 120.23, 115.94, 112.05, 110.42, 98.37, 58.04, 55.97, 36.31, 10.32.


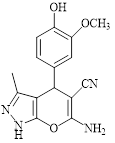


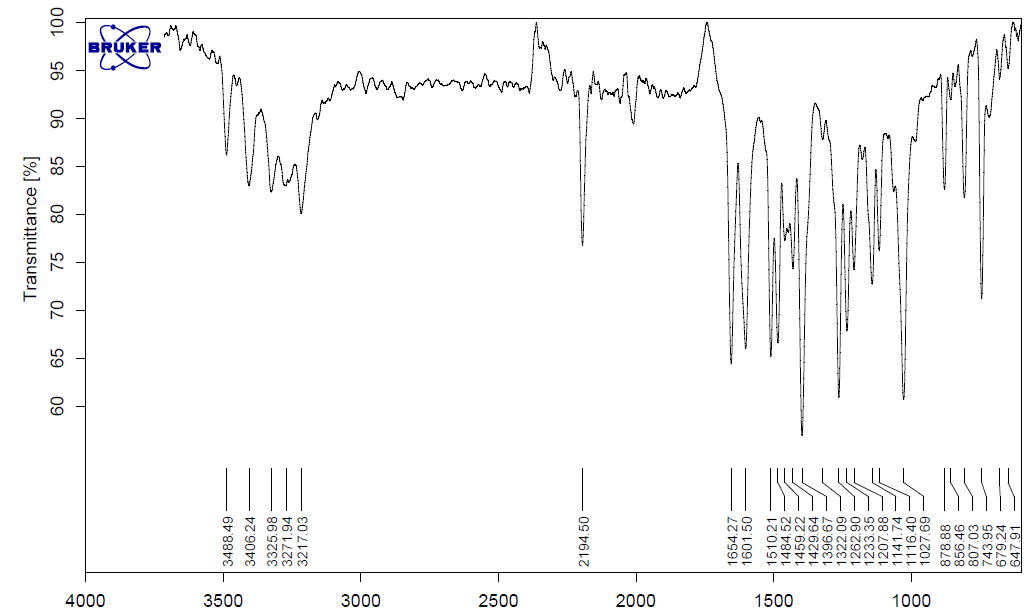


TheFT-IR spectrum of 6-Amino-4-(4-hydroxy-3-methoxyphenyl)-3-methyl-1,4-dihydropyrano[2,3-c]pyrazole-5-carbonitrile


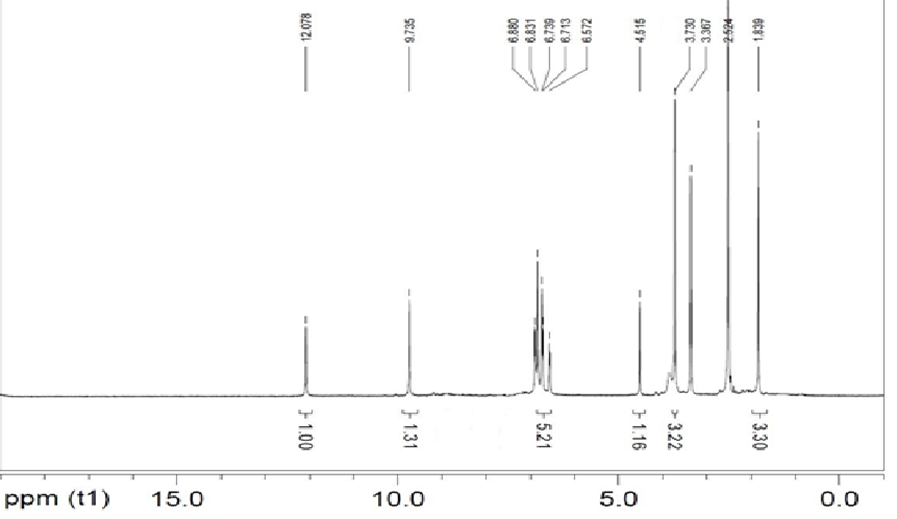


The ^1^H NMR (400MHz) spectrum of 6-Amino-4-(4-hydroxy-3-methoxyphenyl)-3-methyl-1,4-dihydropyrano[2,3-c]pyrazole-5-carbonitrile

**
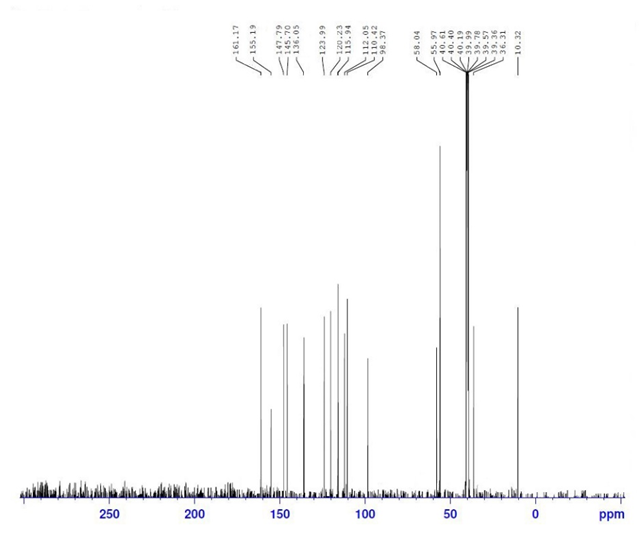
**

The ^13^C NMR (100 MHz) spectrum of 6-Amino-4-(4-hydroxy-3-methoxyphenyl)-3-methyl-1,4-dihydropyrano[2,3-c]pyrazole-5-carbonitrile

*6-Amino-4-(furan-2-yl)-3-methyl-1,4-dihydropyrano[2,3-c]pyrazole-5-carbonitrile .*

Cream solid. M.p. 231-234 °C. FT- IR (ATR) /ῡ (cm^-1)^ = 3357, 3179, 2190, 1645, 1598, 1492, 1405, 1152, 1048, 1009, 751, 656.; ^1^H NMR (400 MHz, Acetone-d_6_) δ/ ppm: 1.94 (s, 3H), 4.75 (s, 1H), 6.15-6.17 (m, 1H), 6.32-6.34 (m, 1H), 6.92 (br s, 2H), 7.49-7.51 (m, 1H), 12.14 (s, 1H). ^13^C NMR (100 MHz, DMSO-d_6_)/δ ppm: 161.96, 156.19, 155.29, 142.75, 136.31, 121.08, 110.72 106.12, 95.59, 54.43, 30.28, 10.05.

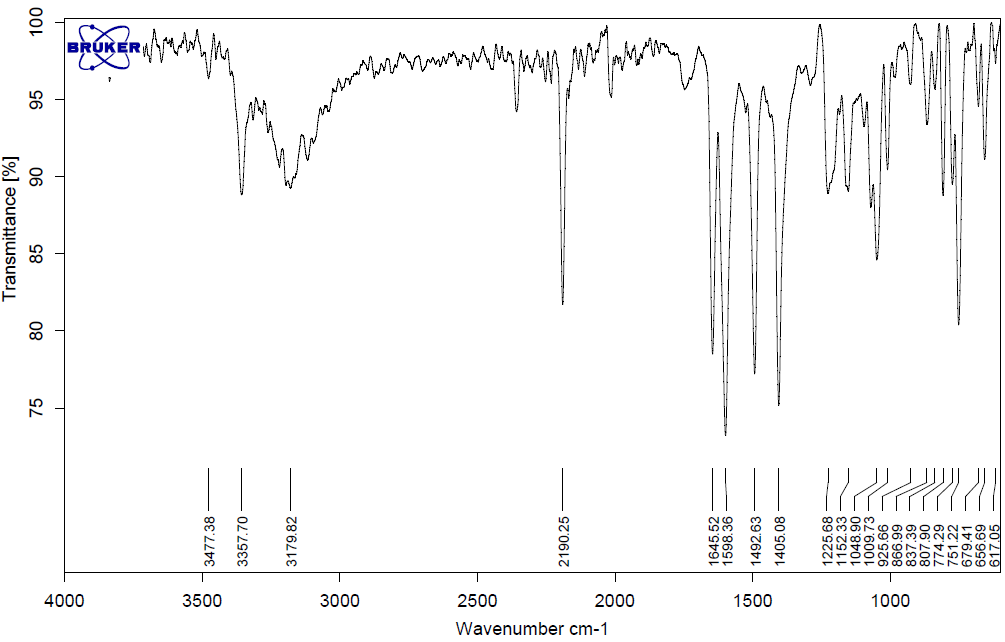


The FT-IR of 6-Amino-4-(furan-2-yl)-3-methyl-1,4-dihydropyrano[2,3-*c*]pyrazole-5-carbonitrile


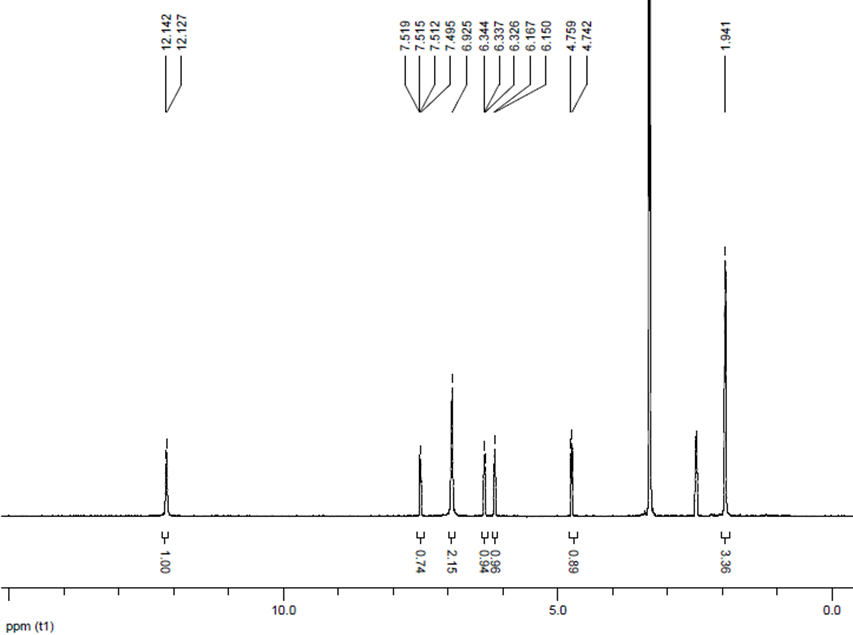


The ^1^H NMR (400 MHz) spectrum of 6-Amino-4-(furan-2-yl)-3-methyl-1,4-dihydropyrano[2,3-*c*]pyrazole-5-carbonitrile


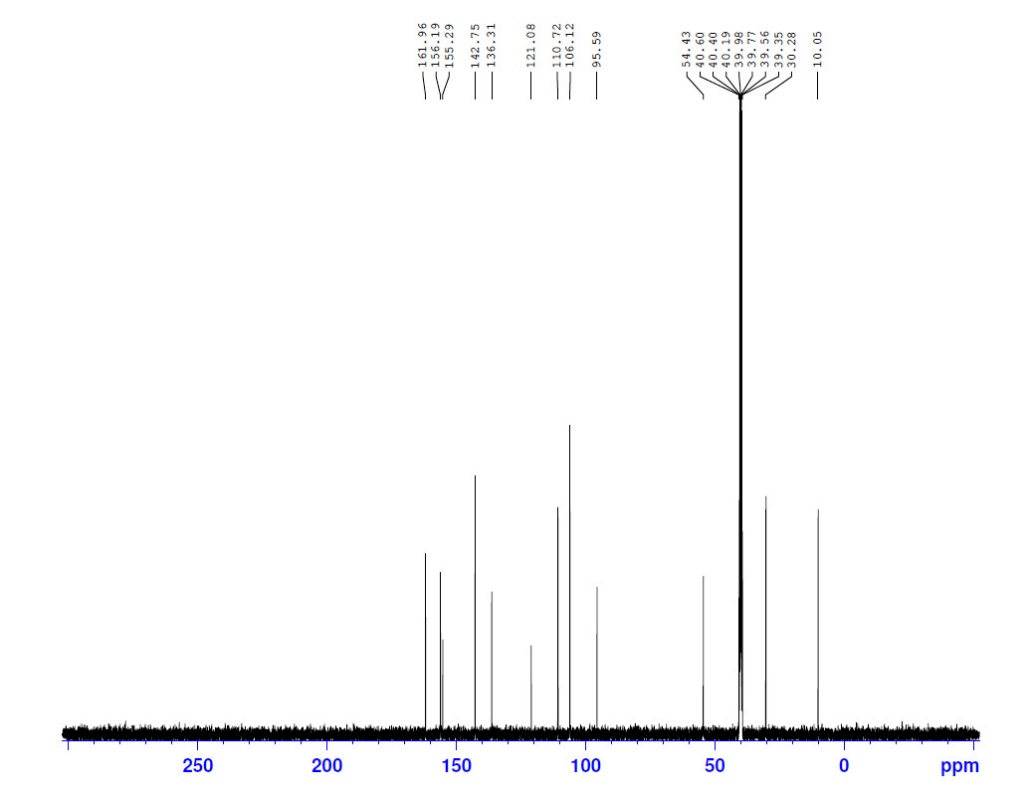


The ^13^C NMR (100 MHz) spectrum of 6-Amino-4-(furan-2-yl)-3-methyl-1,4-dihydropyrano[2,3-*c*]pyrazole-5-carbonitrile

**2-Amino-4-(4-nitrophenyl)-5-oxo-4,5-dihydropyrano[3,2-*c*]chromene-3-carbonitrile**

Pale yellow solid, Melting point: 256-259 ºC, FT-IR (ATR)/υ(cm^-1^): 3429, 3369, 3334, 2195, 1716, 1672, 1603. ^1^HNMR (400MHz, DMSO-d_6_) δ (ppm): 4.67 (s, 1H, CH), 8.17 (d, 2H, *J*=8.8 Hz, Ar-H), 7.91 (d, 1H, *J*=7.2 Hz, Ar-H), 7.74 (t, 1H, *J*=7.6 Hz, Ar-H), 7.46-4.60 (m, 6H, Ar-H).

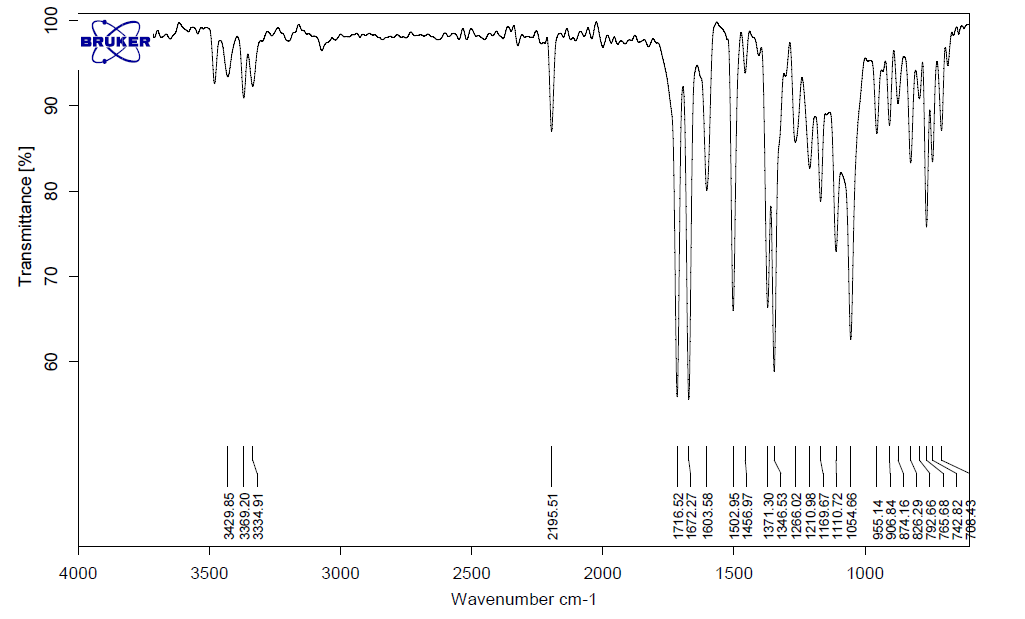


**The FT-IR of** **2-Amino-4-(4-nitrophenyl)-5-oxo-4,5-dihydropyrano[3,2-*c*]chromene-3-carbonitrile**

**
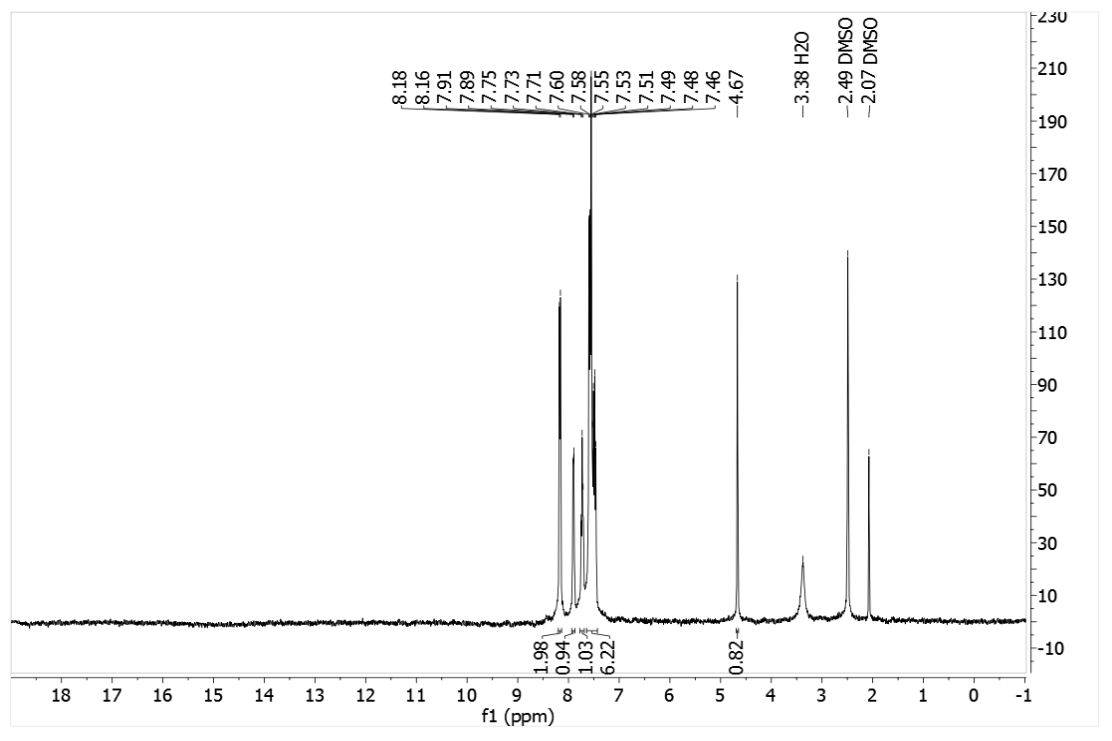
**

**The ^1^H NMR spectrum of 2-Amino-4-(4-nitrophenyl)-5-oxo-4,5-dihydropyrano[3,2-*c*]chromene-3-carbonitrile**

**
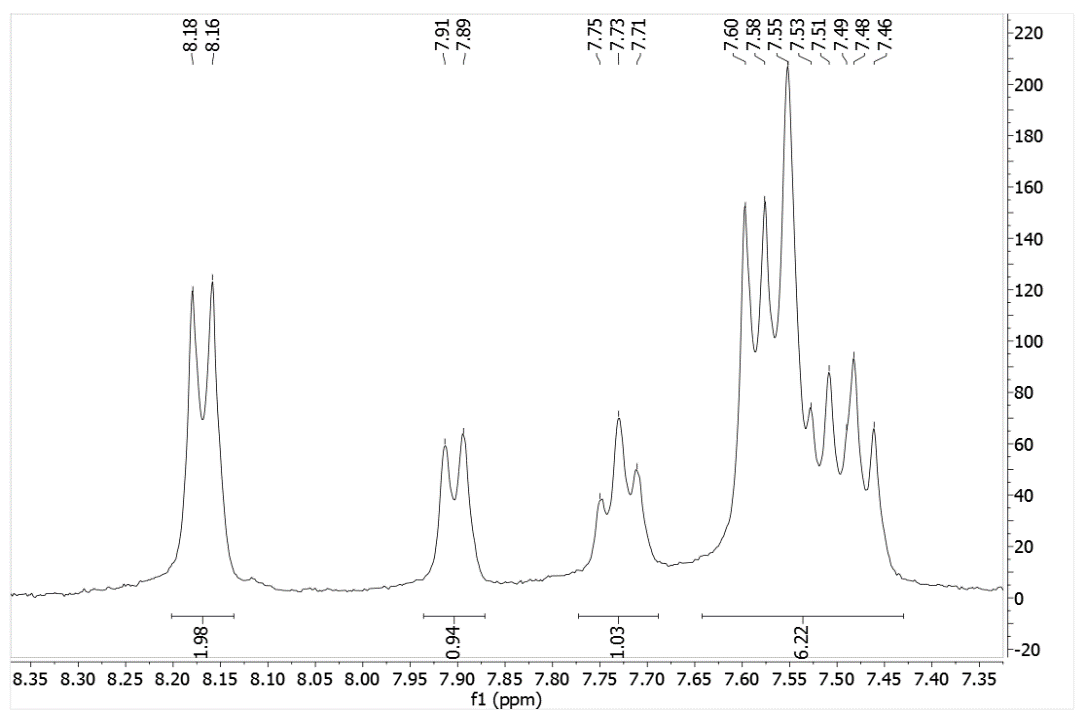
**

**The ^1^H NMR spectrum of 2-Amino-4-(4-nitrophenyl)-5-oxo-4,5-dihydropyrano[3,2-*c*]chromene-3-carbonitrile**

**2-Amino-4-(4-hydroxyphenyl)-5-oxo-4,5-dihydropyrano[3,2-*c*]chromene-3-carbonitrile**

White Solid, Melting point: 266-268 ºC, FT-IR (ATR)/υ(cm^-1^): 3399, 3285, 3180, 2195, 1692, 1670, 1601; ^1^HNMR(500MHz,DMSO-d_6_) δ (ppm): 4.33 (s, 1H), 6.70 (d, 2H, *J* = 8.5 Hz), 7.06 (d, 2H, *J* = 8.5 Hz), 7.34 (s, 2H, NH_2_), 7.42-7.48 (m, 2H), 7.69 (dt, 1H, *J*_1_ = 7.8 Hz, *J*_2_ = 1.6 Hz), 7.89 (dd, 1H, *J*_1_ = 7.8 Hz, *J*_2_ =1.4 Hz), 9.35 (s, 1H, OH). ^13^C NMR (125 MHz, DMSO-d_6_) *δ* (ppm) 58.40, 75.11, 104.50, 113.03, 114.21, 115.12, 116.63, 119.43, 122.79, 124.68, 128.73, 133.73, 152.05, 152.97, 156.49, 157.90, 159.55, 163.91.

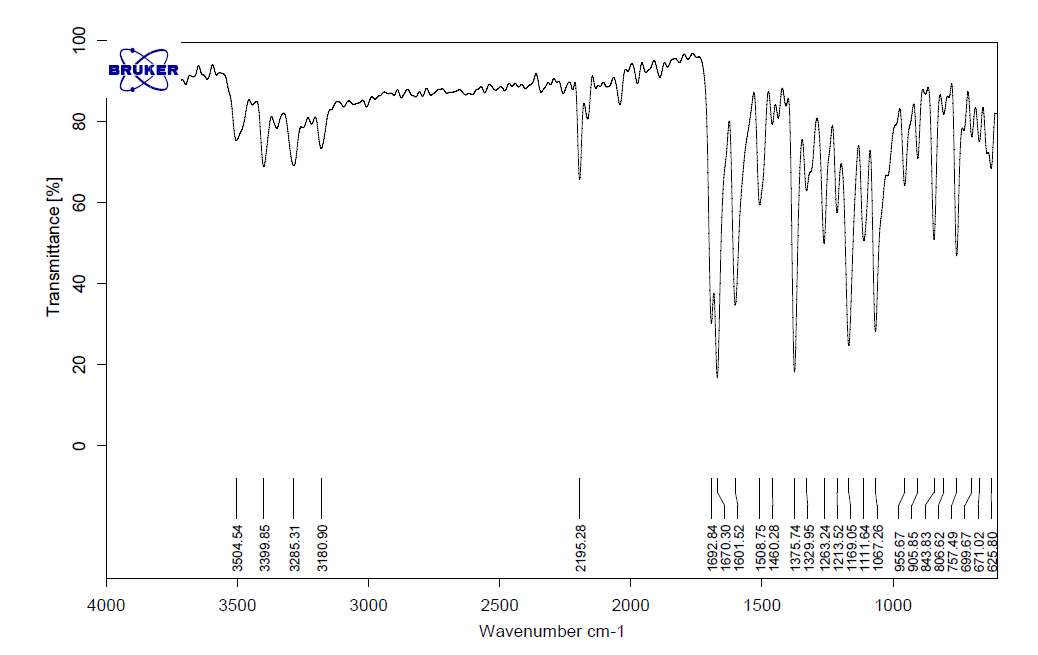


**The FT-IR of 2-Amino-4-(4-hydroxyphenyl)-5-oxo-4,5-dihydropyrano[3,2-*c*]chromene-3-carbonitrile**


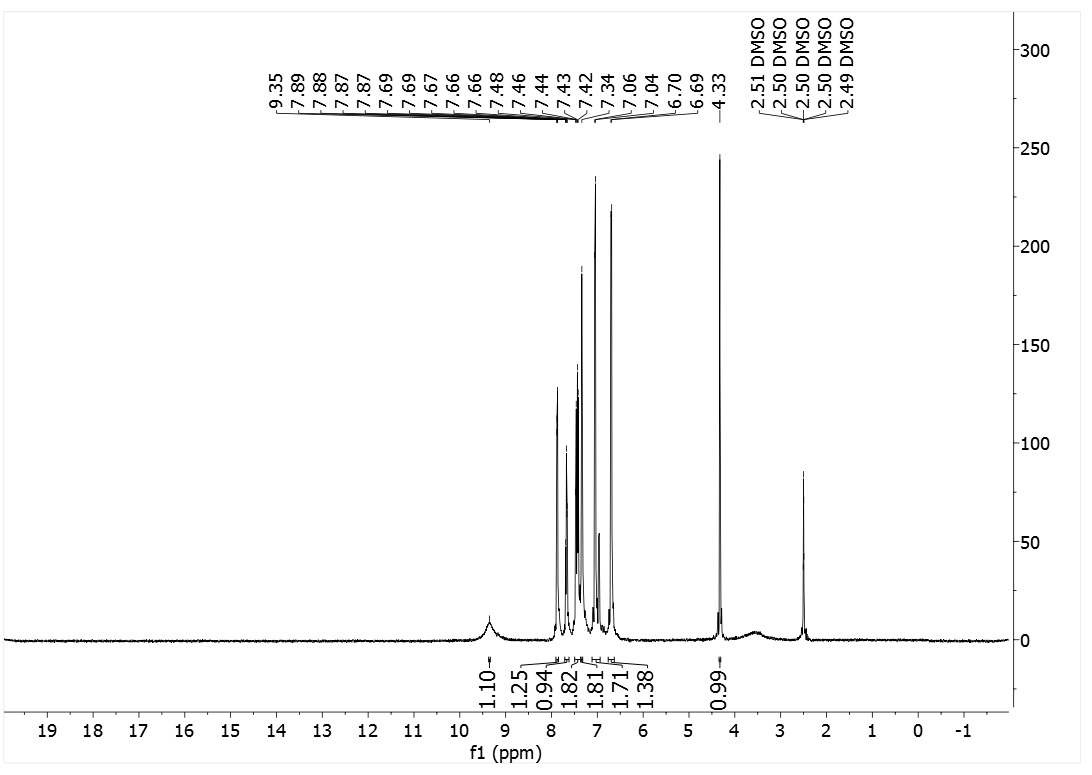


**The ^1^H NMR spectrum of 2-Amino-4-(4-hydroxyphenyl)-5-oxo-4,5-dihydropyrano[3,2-*c*]chromene-3-carbonitrile**


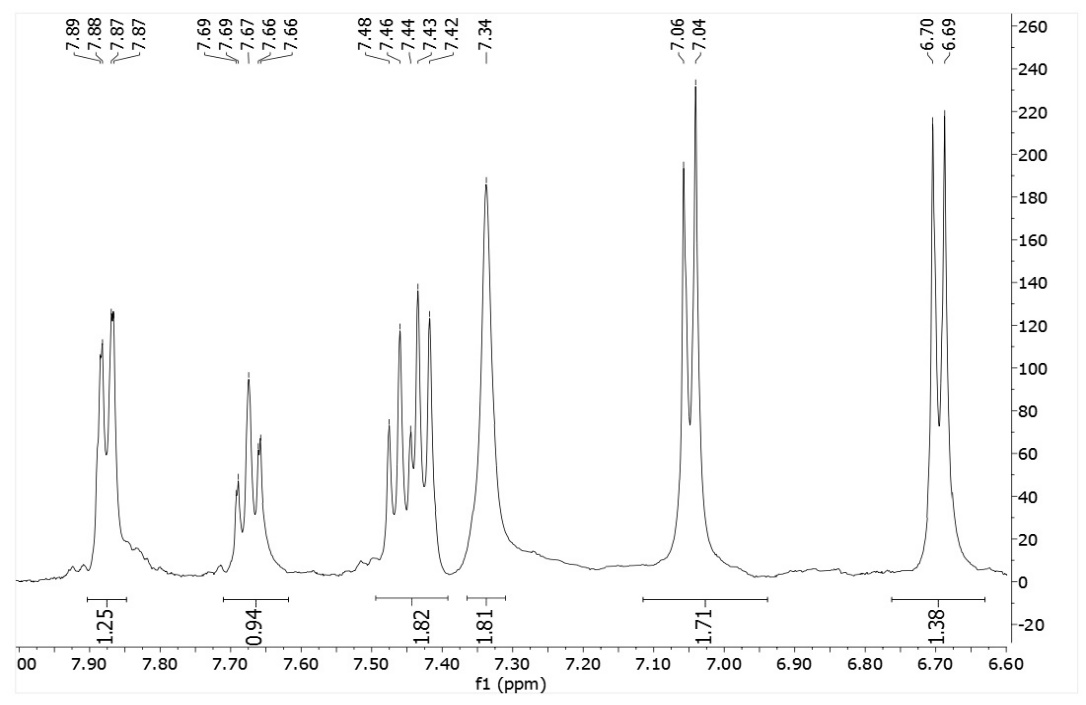


**The ^1^H NMR spectrum of 2-Amino-4-(4-hydroxyphenyl)-5-oxo-4,5-dihydropyrano[3,2-*c*]chromene-3-carbonitrile**


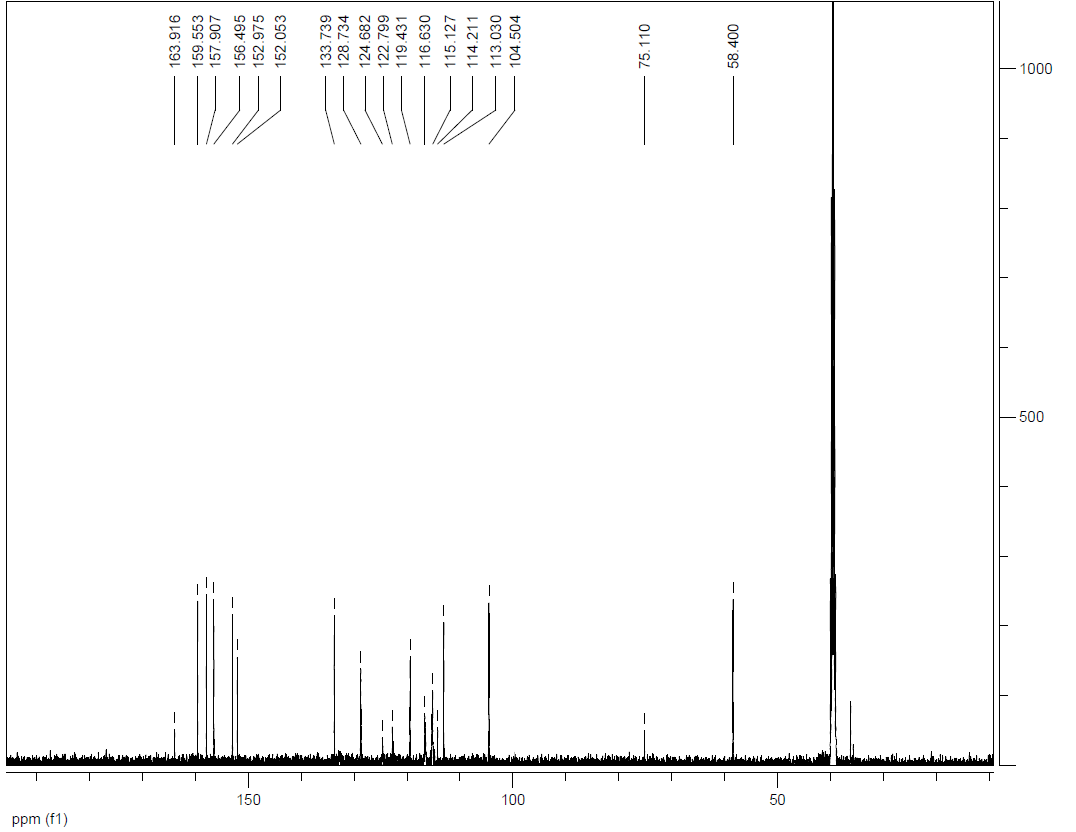


**The ^13^C NMR spectrum of 2-Amino-4-(4-hydroxyphenyl)-5-oxo-4,5-dihydropyrano[3,2-*c*]chromene-3-carbonitrile**

**2-Amino-4-(3-nitrophenyl)-5-oxo-4,5-dihydropyrano[3,2-*c*]chromene-3-carbonitrile**

Yellow solid, Melting point: 260-263 ºC, FT-IR (ATR)/υ(cm^-1^): 3401, 3317, 3191, 2202, 1700, 1668, 1605, 1527, 1380, 1207, 1112, 1055, 956, 733; ^1^HNMR (400MHz, DMSO-d_6_) δ( ppm): 4.73 (1H,s, CH),7.45-7.52 (2H, m, Ar-H), 7.57 (2H, s, NH_2_), 7.61-7.65 (1H, m, Ar-H), 7.72 (1H, td, *J_1_*=1.6 Hz, *J*_2_=8Hz, Ar-H), 7.81 (1H, d, *J*=8Hz, Ar-H), 7.92 (1H, dd, *J*_1_=1.6Hz, *J_2_*=8Hz, Ar-H), 8.11 (2H, d, *J*=8Hz, Ar-H)

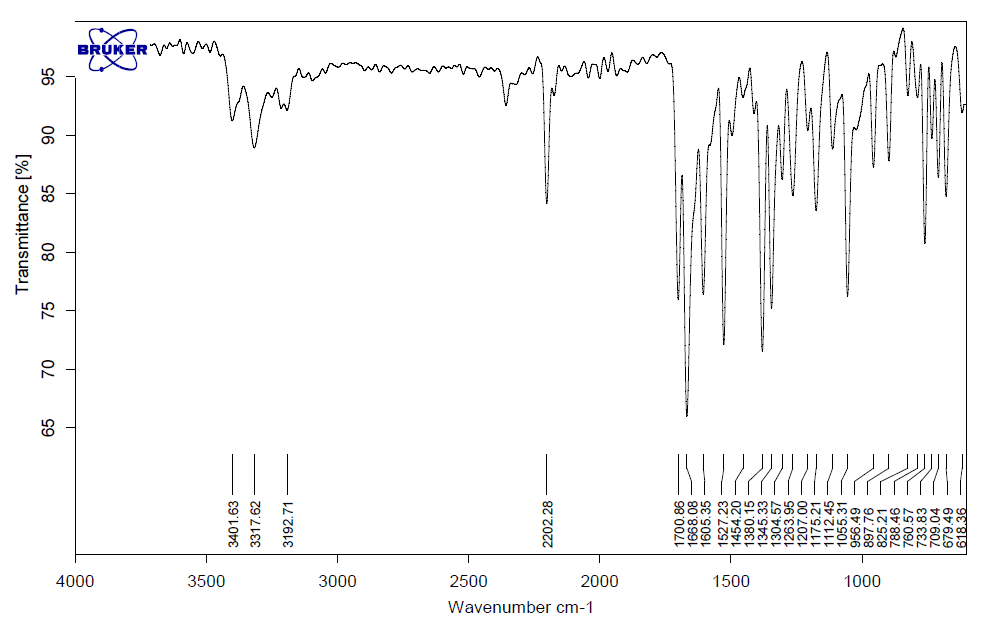


**The FT-IR of 2-Amino-4-(3-nitrophenyl)-5-oxo-4,5-dihydropyrano[3,2-*c*]chromene-3-carbonitrile**


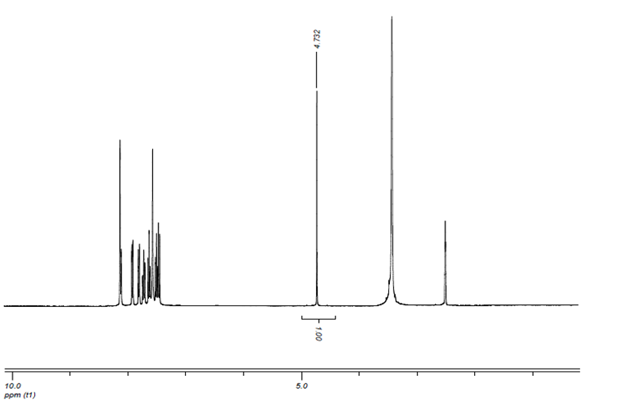


**The ^1^H NMR spectrum of 2-Amino-4-(3-nitrophenyl)-5-oxo-4,5-dihydropyrano[3,2-*c*]chromene-3-carbonitrile**


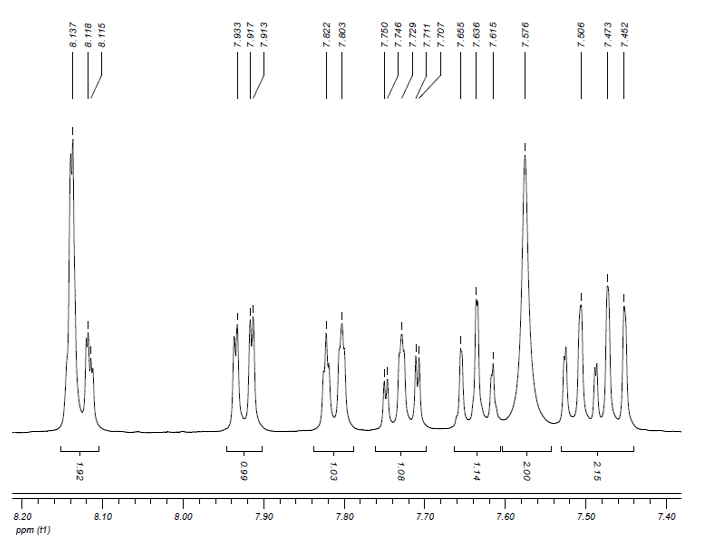


**The ^1^H NMR spectrum of 2-Amino-4-(3-nitrophenyl)-5-oxo-4,5-dihydropyrano[3,2-*c*]chromene-3-carbonitrile**

**2-Amino-4-(2-nitrophenyl)-5-oxo-4,5-dihydropyrano[3,2-*c*]chromene-3-carbonitrile**

Pale yellow solid, Melting point: 241-243 ºC, FT-IR (ATR)/υ(cm^-1^): 3398, 3287, 3183, 2195, 1700, 1670, 1602; ^1^HNMR(500MHz, DMSO-d_6_) δ( ppm): 5.24 (s, 1H), 7.44-7.57 (m, 6H), 7.66 (t, 1H, *J* = 7.5 Hz), 7.73 (dt, 1H, *J* = 7.5 Hz), 7.90 (d, 2H, *J* = 8 Hz). ^13^C NMR (125MHz, DMSO-d6) *δ* (ppm) 31.56, 56.02, 103.32, 112.84, 116.67, 118.72, 122.48, 123.90, 124.08, 124.87, 131.29, 133.48, 137.36, 149.19, 152.18, 153.59, 158.61, 159.74.

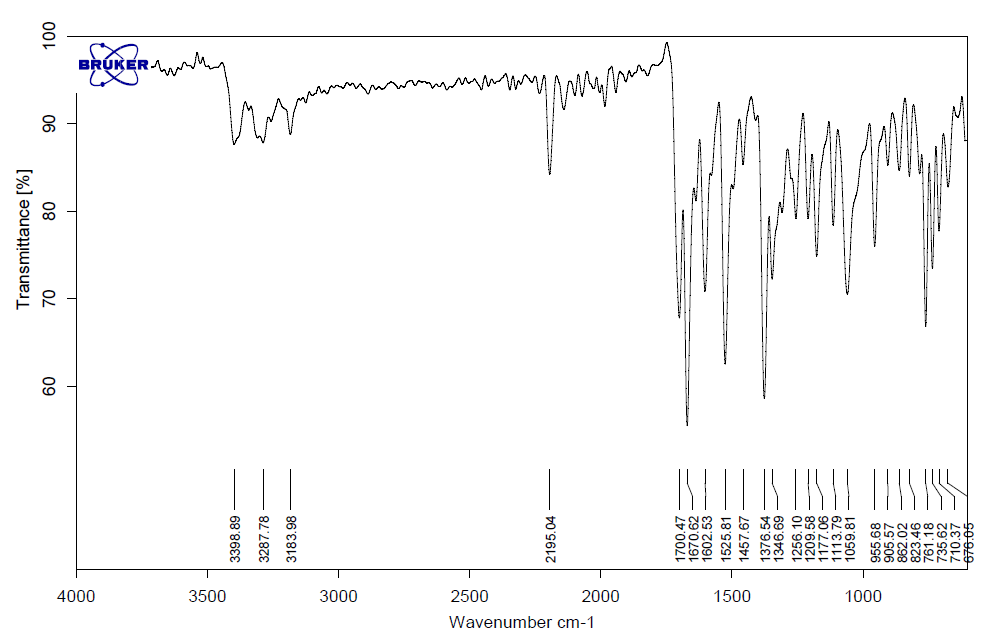


**The FT-IR of 2-Amino-4-(2-nitrophenyl)-5-oxo-4,5-dihydropyrano[3,2-*c*]chromene-3-carbonitrile**


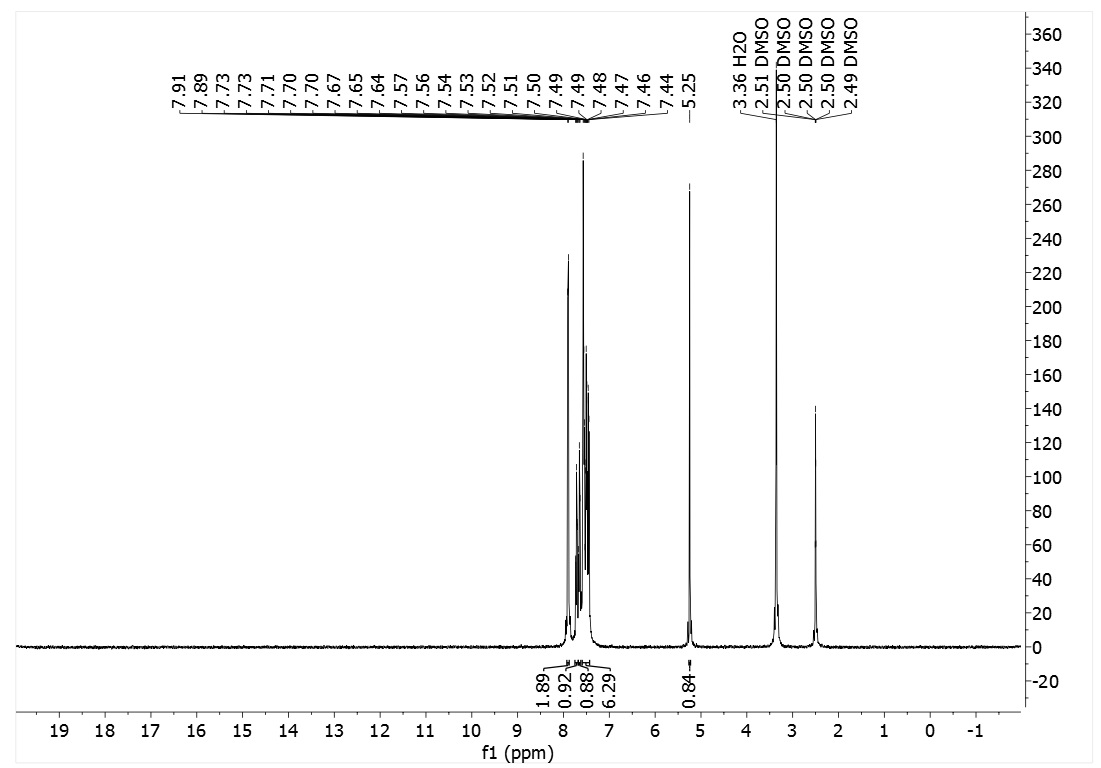


**The ^1^H NMR spectrum of 2-Amino-4-(2-nitrophenyl)-5-oxo-4,5-dihydropyrano[3,2-*c*]chromene-3-carbonitrile**


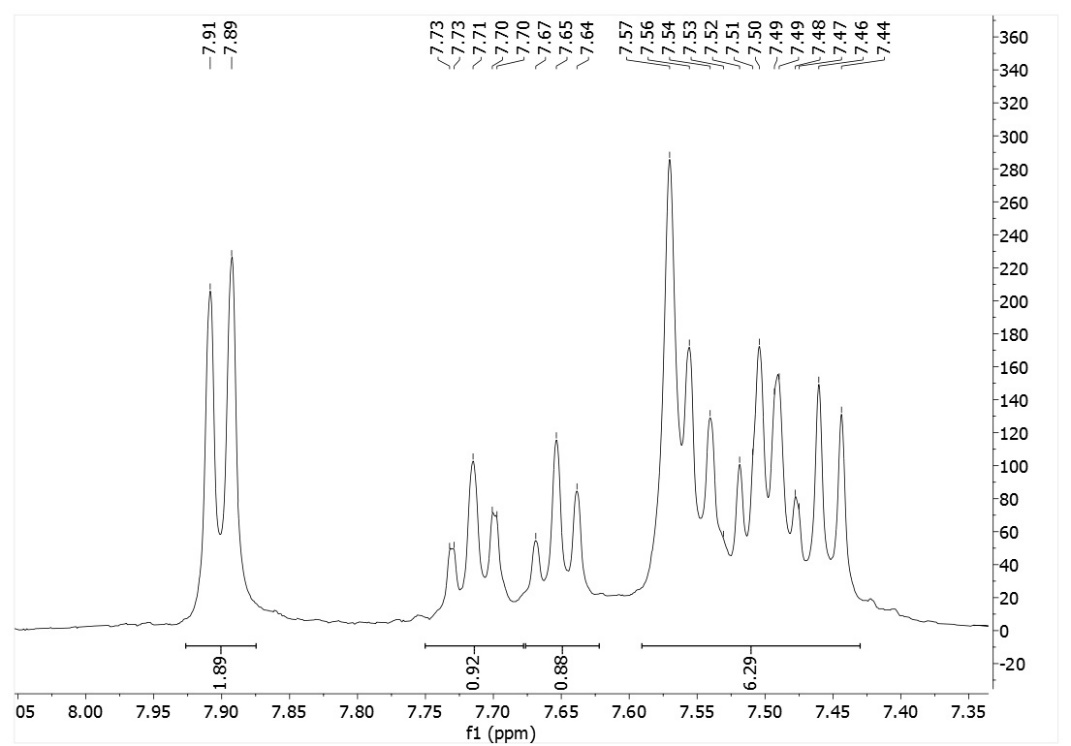


**The ^1^H NMR spectrum of 2-Amino-4-(2-nitrophenyl)-5-oxo-4,5-dihydropyrano[3,2-*c*]chromene-3-carbonitrile**


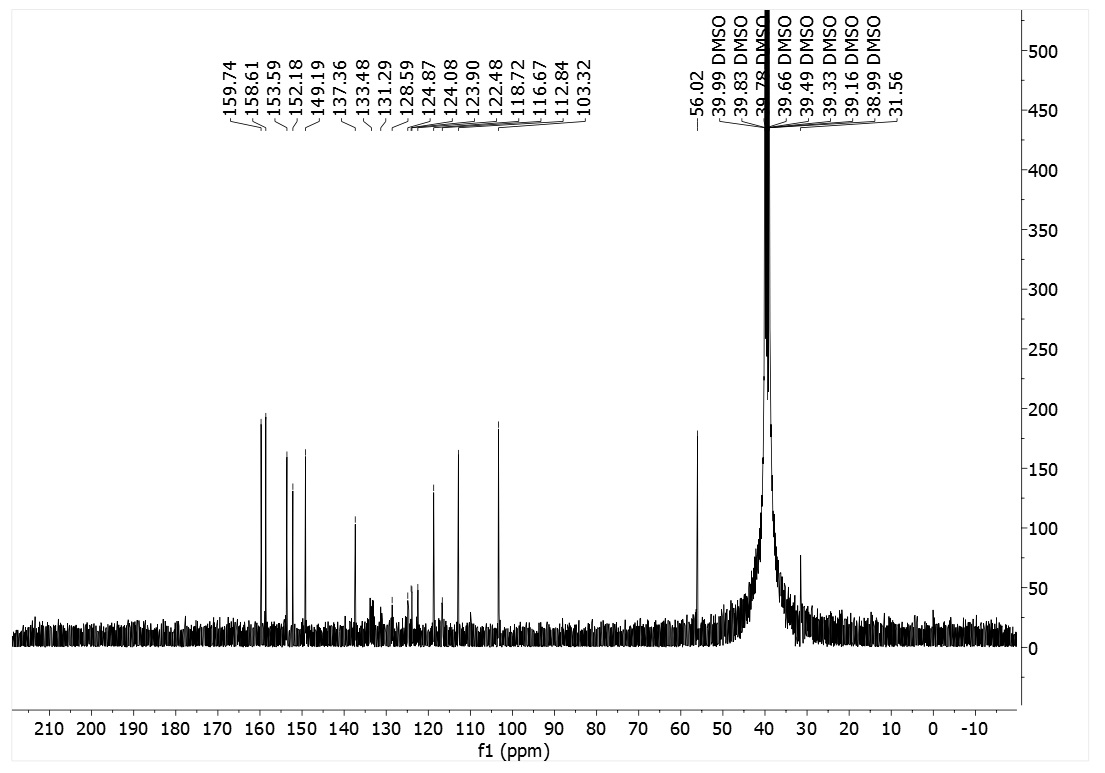


**The ^13^C NMR spectrum of 2-Amino-4-(2-nitrophenyl)-5-oxo-4,5-dihydropyrano[3,2-*c*]chromene-3-carbonitrile**

**2-Amino-4-(2-chlorophenyl)-5-oxo-4,5-dihydropyrano[3,2-*c*]chromene-3-carbonitrile**

White Solid, Melting point: 262-264 ºC**,** FT-IR (ATR)/υ(cm^-1^): 3392, 3281, 3175, 2200, 1704, 1671, 1601; ^1^HNMR (500MHz, DMSO-d_6_) δ( ppm): 4.98 (1H, s, CH ), 7.26-7.33 (m, 3H, Ar-H), 7.40-7.52 (m, 5H, Ar-H, NH_2_), 7.65-7.74 (m, 2H, Ar-H), 7.90-7.92 (dd, 1H, *J_1_*=1Hz, *J_2_*=7.5Hz, Ar-H);^13^C NMR (125 MHz, DMSO-d_6_) δ( ppm): 56.44, 86.64, 102.92, 112.21, 112.82, 113.47, 118.84, 127.72, 128.82, 129.63, 132.40, 134.28, 140.21, 152.20, 154.04, 158.03, 158.13, 159.42.

**
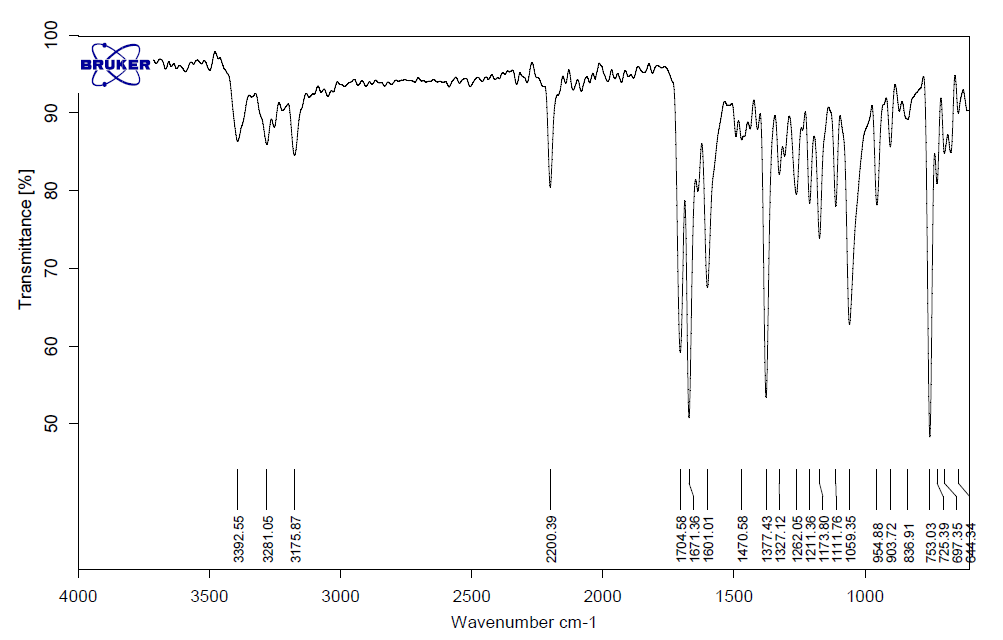
**

**The FT-IR of 2-Amino-4-(2-chlorophenyl)-5-oxo-4,5-dihydropyrano[3,2-*c*]chromene-3-carbonitrile**


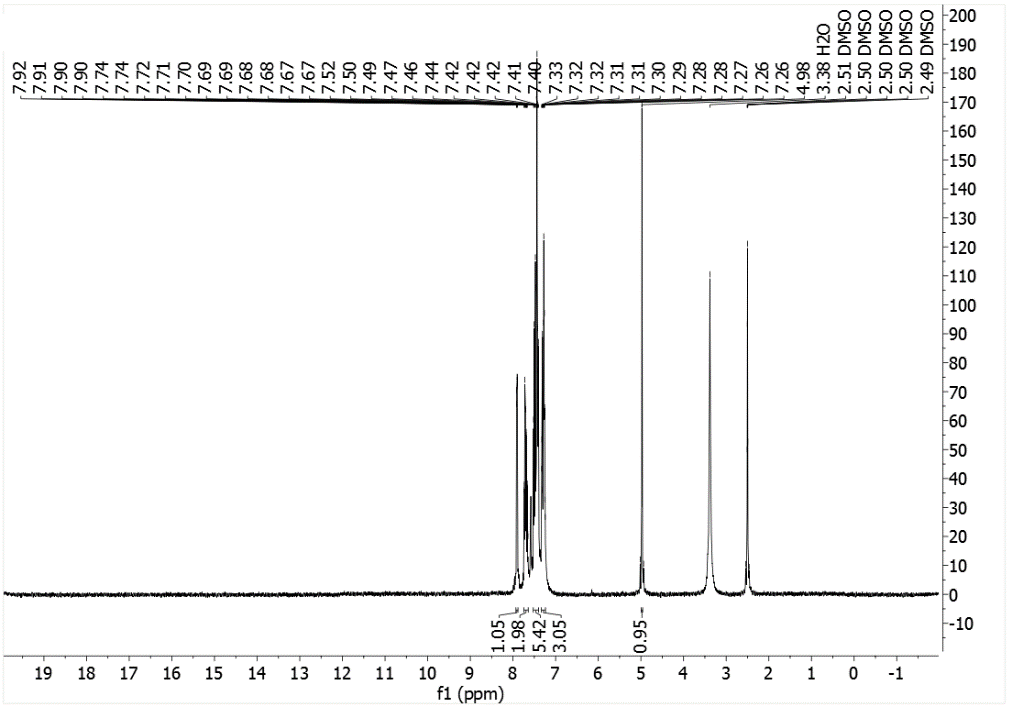


**The ^1^H NMR spectrum of 2-Amino-4-(2-chlorophenyl)-5-oxo-4,5-dihydropyrano[3,2-*c*]chromene-3-carbonitrile**


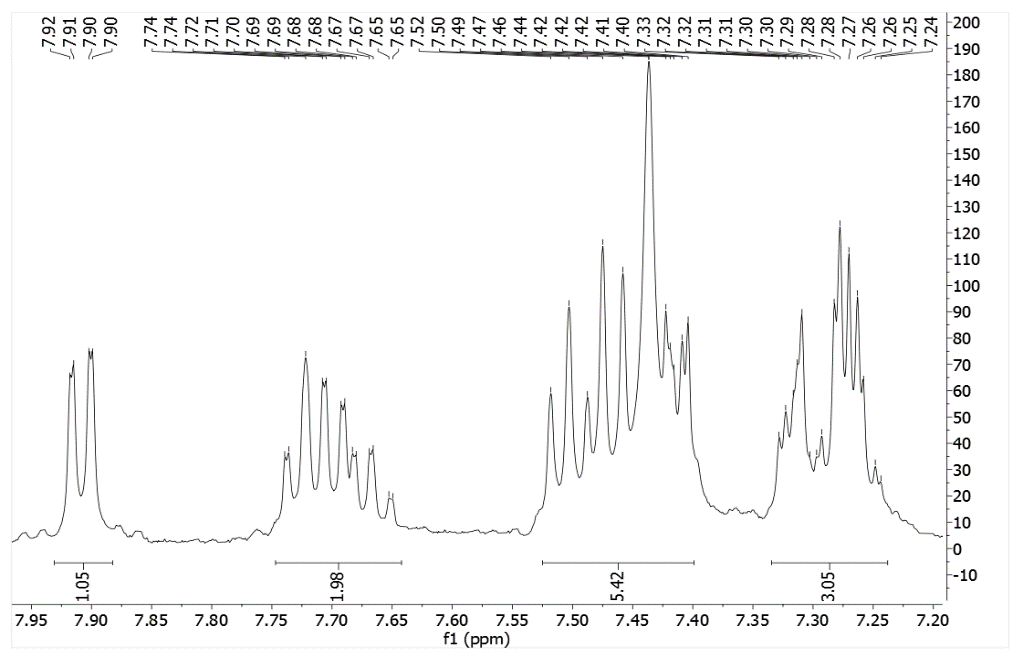


**The ^1^H NMR spectrum of 2-Amino-4-(2-chlorophenyl)-5-oxo-4,5-dihydropyrano[3,2-*c*]chromene-3-carbonitrile**


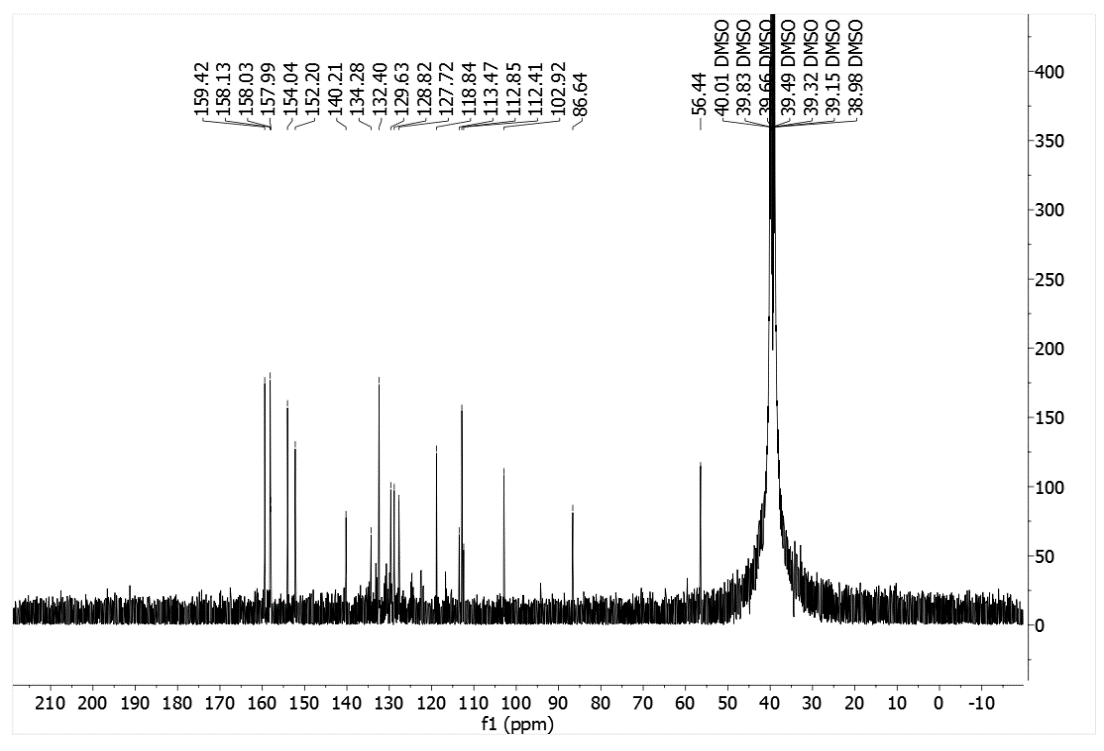


**The ^13^C NMR spectrum of 2-Amino-4-(2-chlorophenyl)-5-oxo-4,5-dihydropyrano[3,2-*c*]chromene-3-carbonitrile**

**2-Amino-4-(4-methoxyphenyl)-5-oxo-4,5-dihydropyrano[3,2-*c*]chromene-3-carbonitrile**

Pale yellow solid, Melting point: 247-250 ºC, FT-IR (ATR)/υ(cm^-1^): 3401, 3322, 3217, 2194, 1707, 1664, 1596; ^1^HNMR(500MHz,DMSO-d_6_) δ (ppm): 3.71 (s, 3H, OCH_3_), 4.41 (s, 1H), 6.75-6.73 (dd, 1H, *J*_1_ = 8.5 Hz, *J*_2_ = 2.5 Hz, Ar-H), 6.84-6.88 (m, 2H, Ar-H), 7.37 (s, 2H, NH_2_), 7.45-7.50 (m, 2H, Ar-H), 7.72 (t, 1H, *J* = 7Hz, Ar-H), 7.88-7.90 (dd, 1H, *J*_1_ = 8 Hz, *J*_2_ =1.5 Hz, Ar-H). ^13^C NMR (125 MHz, DMSO-d_6_) *δ* (ppm) 55.49, 58.11, 66.34, 78.97, 101.95, 104.11, 106.59, 112.22, 113.05, 116.66, 119.34, 119.67, 124.69, 135.84, 147.93, 148.50, 152.11, 153.18, 157.92, 159.60.

**
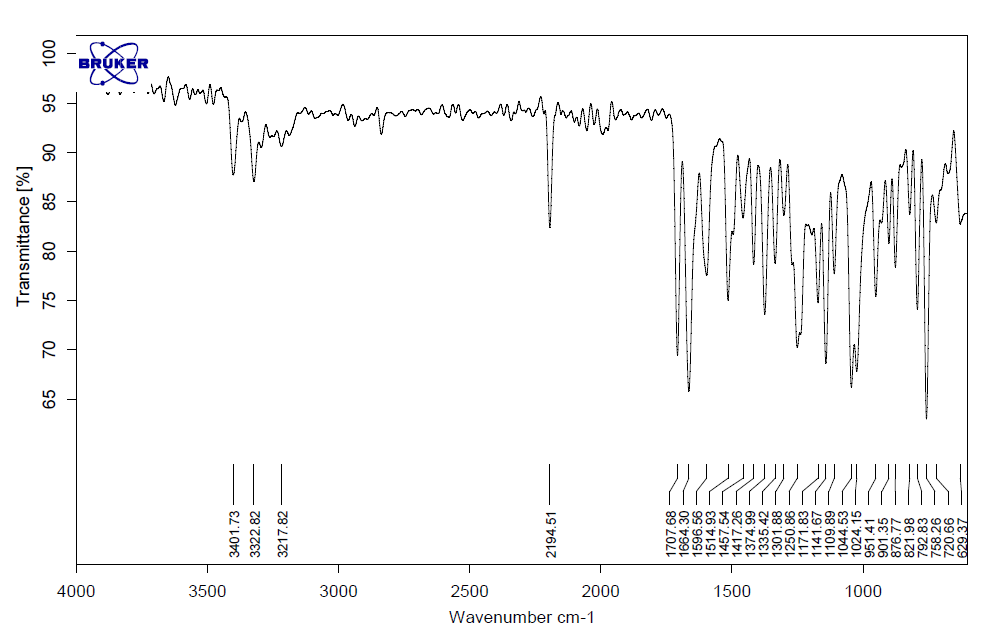
**

**The FT-IR of 2-Amino-4-(4-methoxyphenyl)-5-oxo-4,5-dihydropyrano[3,2-*c*]chromene-3-carbonitrile**

**
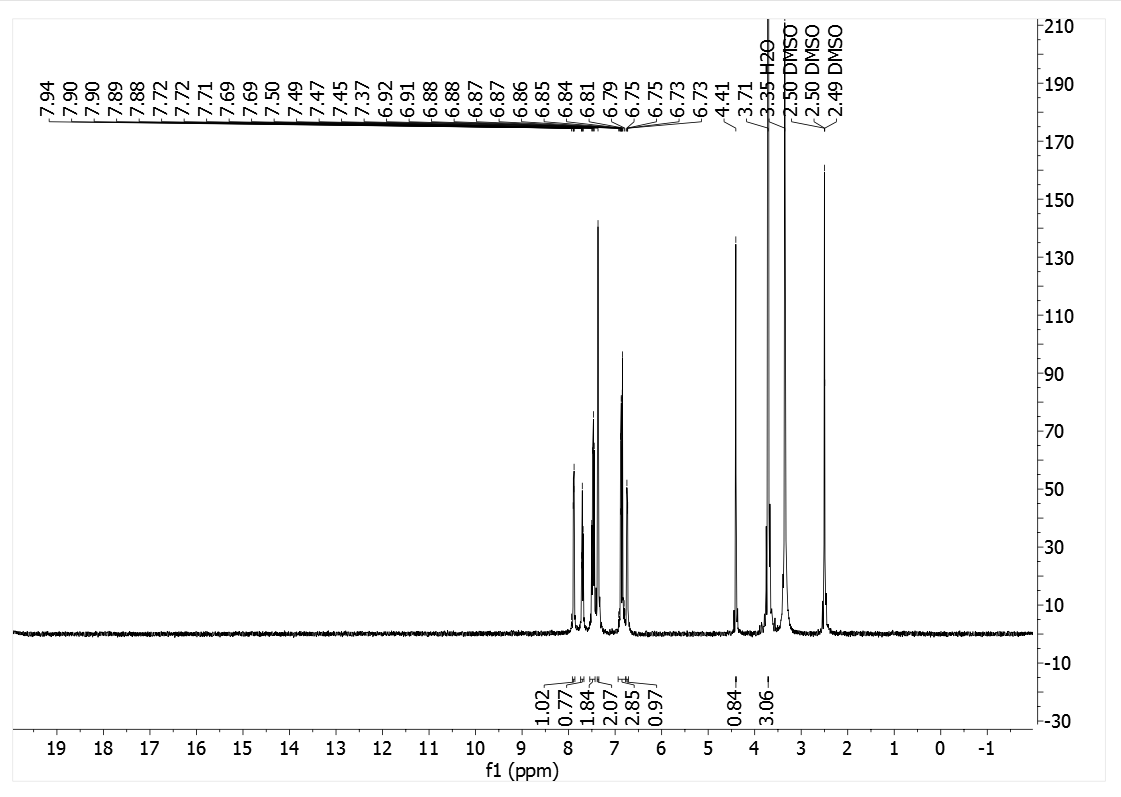
**

**The ^1^H NMR spectrum of 2-Amino-4-(4-methoxyphenyl)-5-oxo-4,5-dihydropyrano[3,2-*c*]chromene-3-carbonitrile**

**
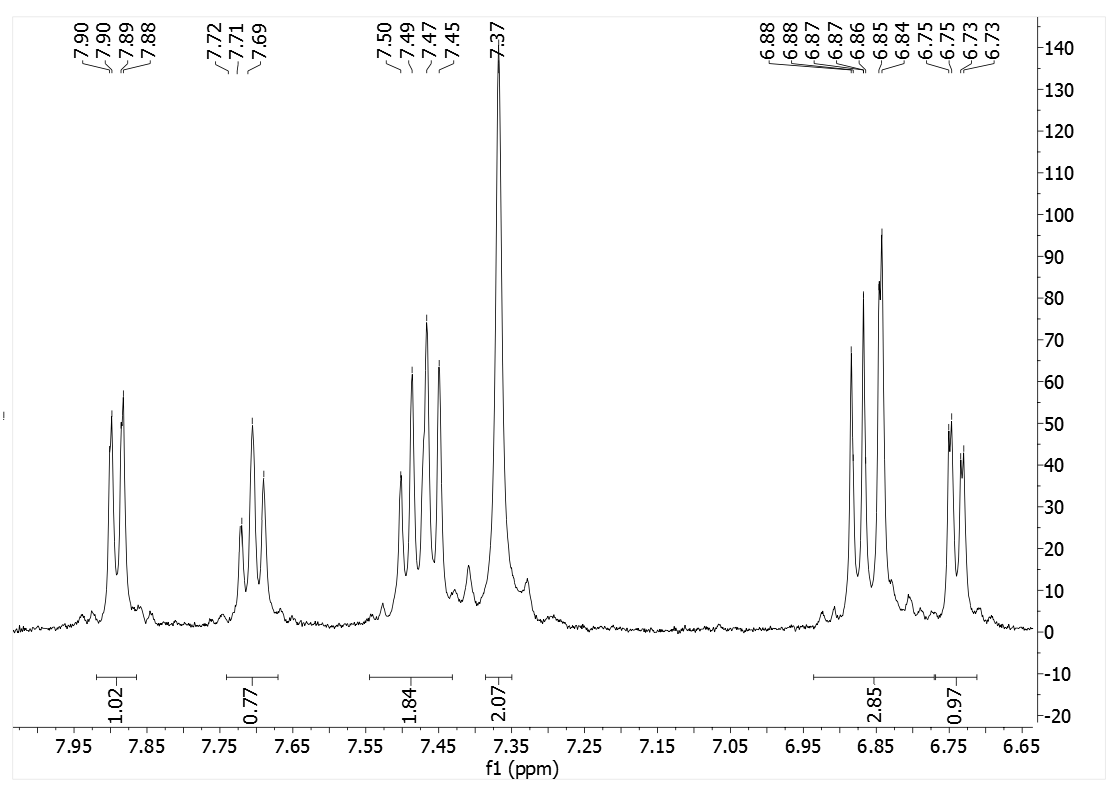
**

**The ^1^H NMR spectrum of 2-Amino-4-(4-methoxyphenyl)-5-oxo-4,5-dihydropyrano[3,2-*c*]chromene-3-carbonitrile**

**
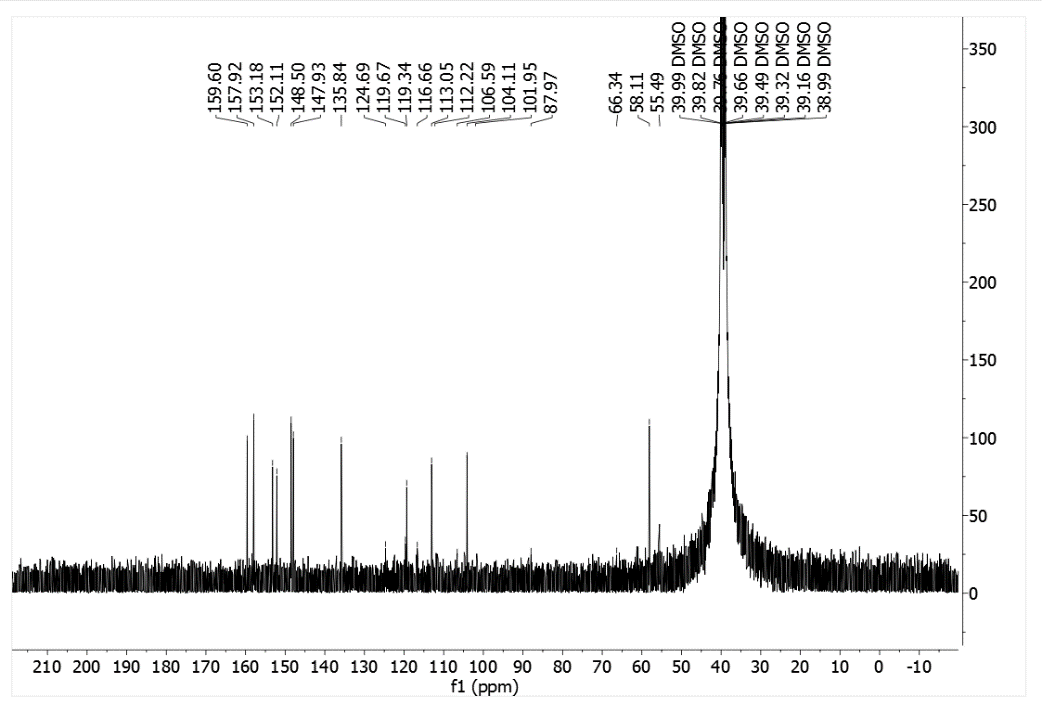
**

**The ^13^C NMR spectrum of 2-Amino-4-(4-methoxyphenyl)-5-oxo-4,5-dihydropyrano[3,2-*c*]chromene-3-carbonitrile**

**2-Amino-4-(2,6-dichlorophenyl)-5-oxo-4,5-dihydropyrano[3,2-*c*]chromene-3-carbonitrile** White Solid, Melting point: 253-256 ºC**,** FT-IR (ATR)/υ(cm^-1^): 3415, 3276, 3170, 2200, 1703, 1669, 1633. ^1^HNMR (400MHz, DMSO-d_6_) δ( ppm): 5.52 (d, 1H,CH), 7.29-7.38 (m, 2H, Ar-H), 7.46-7.52 (m, 5H, Ar-H, NH_2_), 7.74 (t, 1H, *J*=8.4 Hz, Ar-H), 7.88 (d, 1H, *J*=7.5, Ar-H).

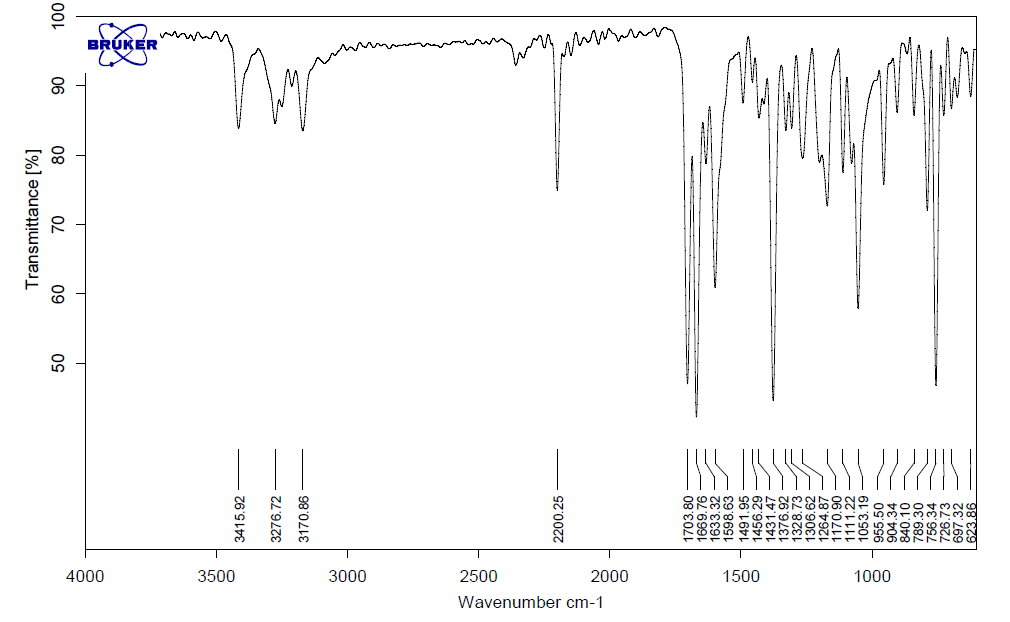


**The FT-IR of 2-Amino-4-(2,6-dichlorophenyl)-5-oxo-4,5-dihydropyrano[3,2-*c*]chromene-3-carbonitrile**


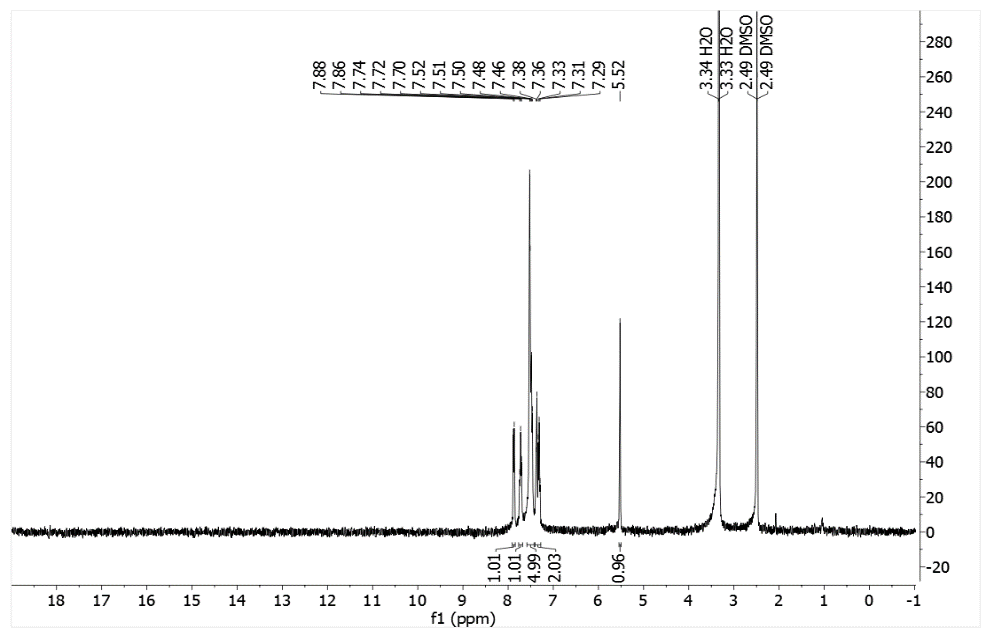


**The ^1^H NMR spectrum of 2-Amino-4-(2,6-dichlorophenyl)-5-oxo-4,5-dihydropyrano[3,2-*c*]chromene-3-carbonitrile**


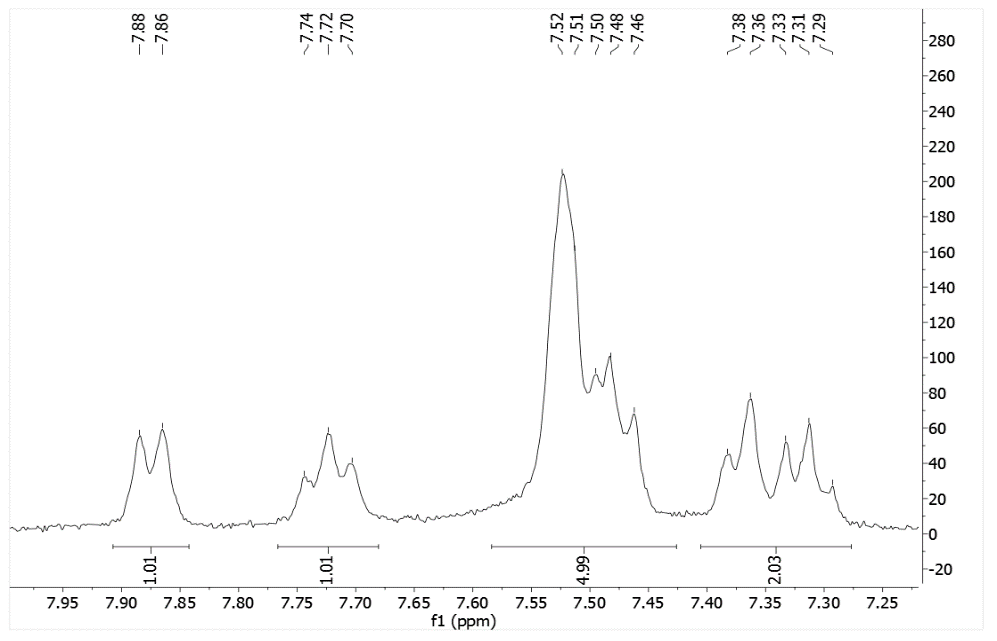


**The ^1^H NMR spectrum of 2-Amino-4-(2,6-dichlorophenyl)-5-oxo-4,5-dihydropyrano[3,2-*c*]chromene-3-carbonitrile**

**2-Amino-5-oxo-4-phenyl-4,5-dihydropyrano[3,2-*c*]chromene-3-carbonitrile**

White Solid, Melting point: 256-258 ºC, FT-IR (ATR)/υ(cm^-1^): 3371, 3284, 3176, 2196, 1706, 1671, 1604. ^1^HNMR (500MHz, DMSO-d_6_) δ( ppm): 4.45 (1H, s, CH ), 7.22-7.33 (5H, m, Ar-H), 7.42-7.49 (4H, m, Ar-H,NH_2_), 7.71 (1H, t, *J=*8Hz, Ar-H), 7.91 (1H, d, *J*=8Hz, Ar-H). ^13^C NMR (125 MHz, DMSO-d_6_) δ( ppm): 37.00, 57.94, 103.99, 112.95, 116.61, 119.28, 122.38, 122.56, 124.77, 127.13, 127.65, 128.53, 132.83, 133.04, 143.36, 152.13, 153.42, 157.98, 159.55.

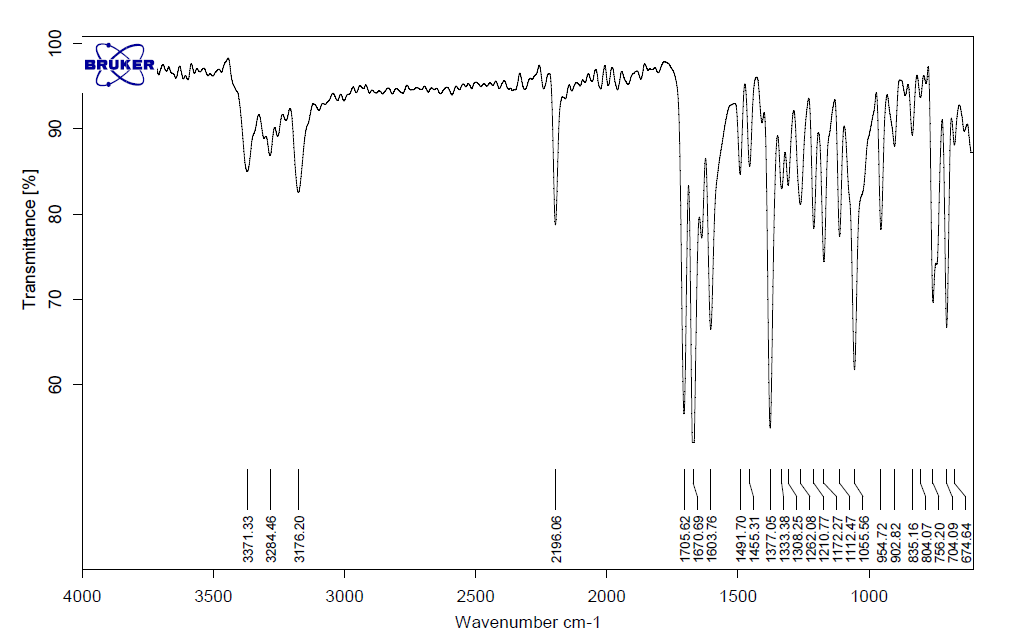


**The FT-IR of 2-Amino-5-oxo-4-phenyl-4,5-dihydropyrano[3,2-*c*]chromene-3-carbonitrile**


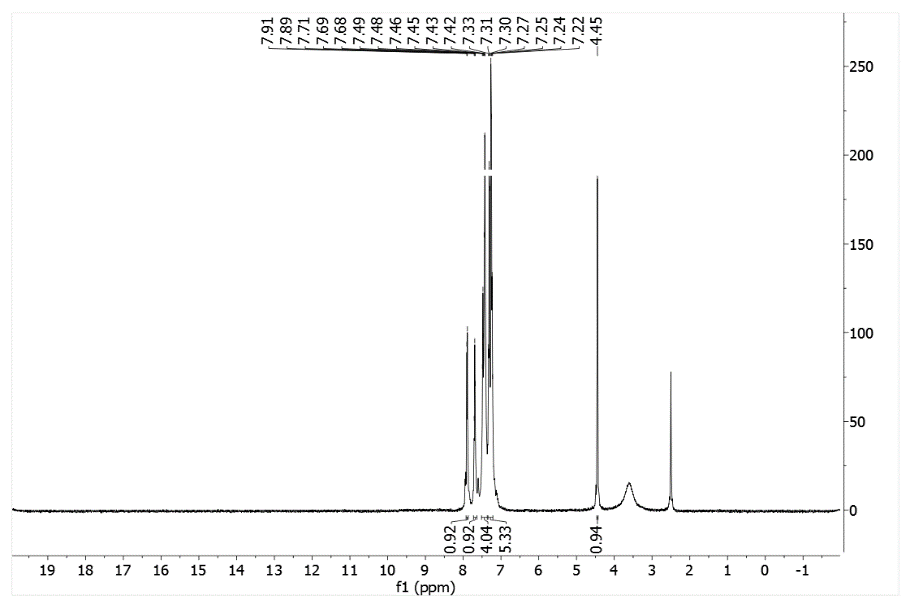


**The ^1^H NMR spectrum of 2-Amino-5-oxo-4-phenyl-4,5-dihydropyrano[3,2-*c*]chromene-3-carbonitrile**


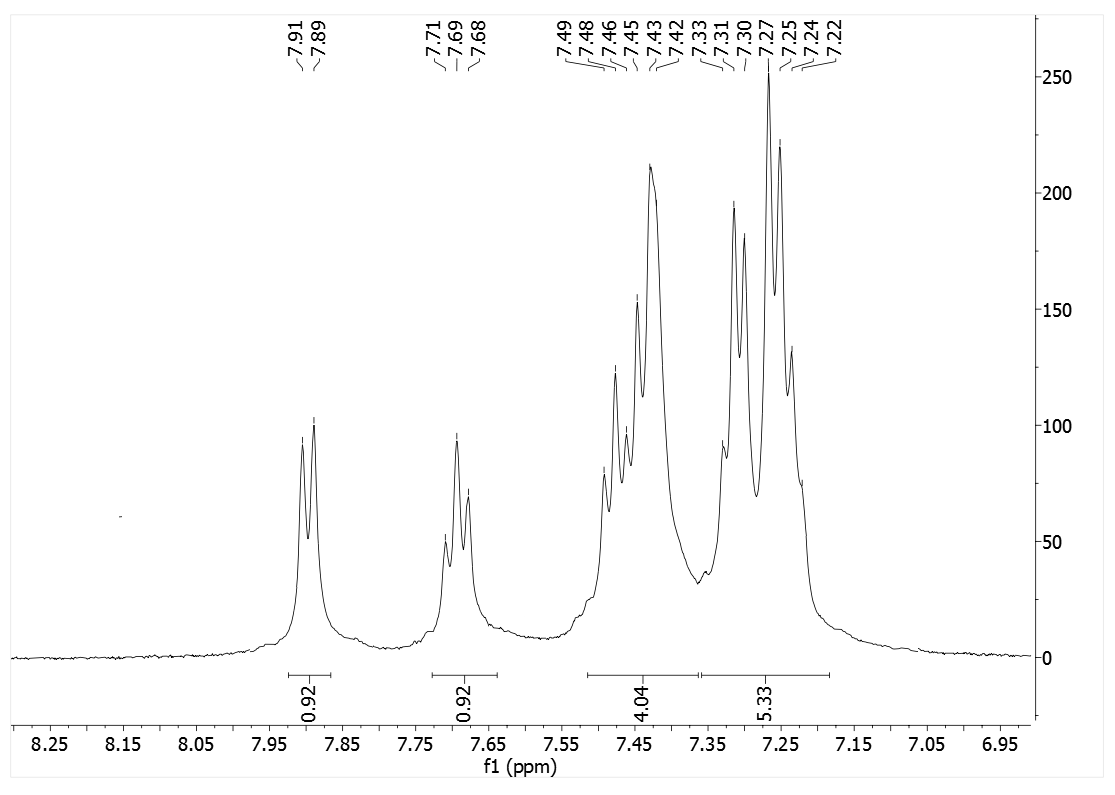


**The ^1^H NMR spectrum of 2-Amino-5-oxo-4-phenyl-4,5-dihydropyrano[3,2-*c*]chromene-3-carbonitrile**


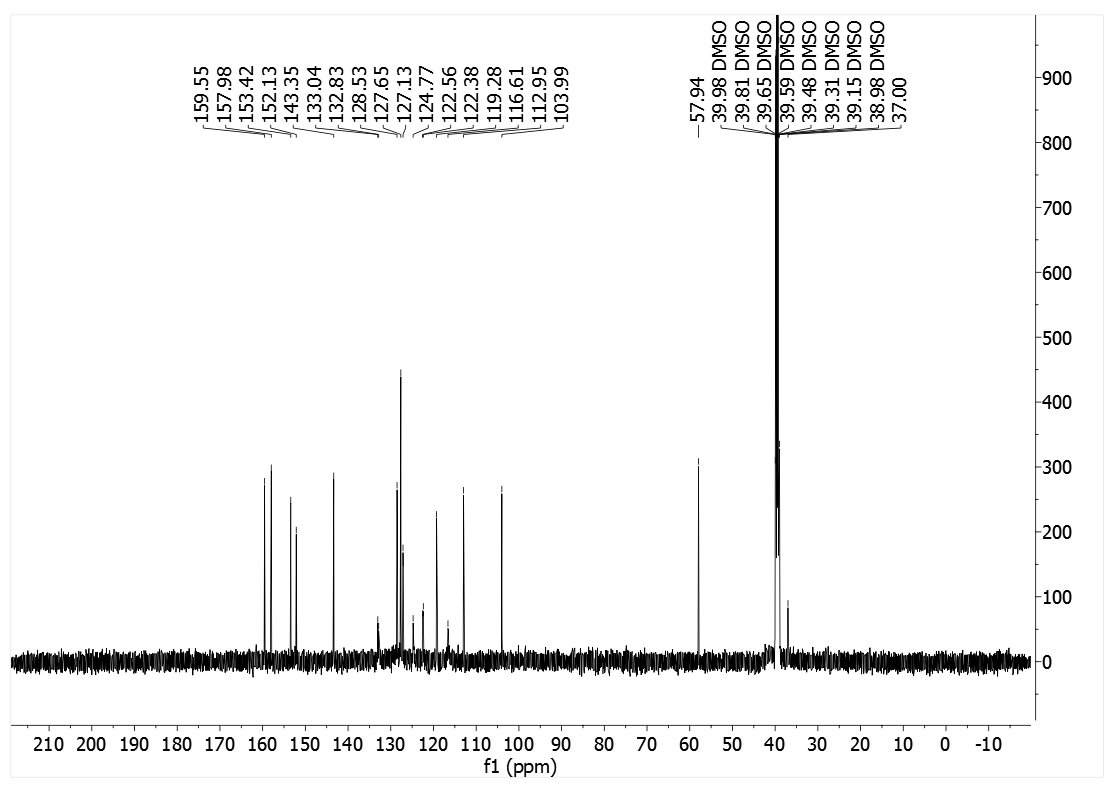


**The ^13^C NMR spectrum of 2-Amino-5-oxo-4-phenyl-4,5-dihydropyrano[3,2-*c*]chromene-3-carbonitrile**

**2-Amino-4-(4-chlorophenyl)-5-oxo-4,5-dihydropyrano[3,2-*c*]chromene-3-carbonitrile**

White Solid, Melting point: 262-265 ºC, FT-IR (ATR)/υ(cm^-1^): 3401, 3320, 3195, 2201, 1700, 1668, 1605; ^1^HNMR (500MHz, DMSO-d_6_) δ( ppm): 4.73 (1H, s, CH ), 7.46 (d, 1H, *J* = 8Hz, Ar-H), 7.51 (t, 1H, *J* = 7.5 , Ar-H), 7.56 (s, 1H, NH_2_), 7.64 (t, 1H, *J*=8Hz, Ar-H), 7.73 (t, 1H, *J* = 8Hz, Ar-H), 7.82 (d, 1H, *J* = 8 Hz, Ar-H), 7.92 (d, 1H, *J* = 7.5 Hz, Ar-H), 8.11-8.14 (m, 2H, Ar-H); ^13^C NMR (125 MHz, DMSO-d_6_) δ( ppm): 36.70, 56.97, 102.93, 113.01, 116.55, 119.04 , 122.31, 122.57, 125.02, 130.26, 132.73, 134.70, 145.56,147.65, 147.90, 152.33, 153.94, 158.20, 159.67.

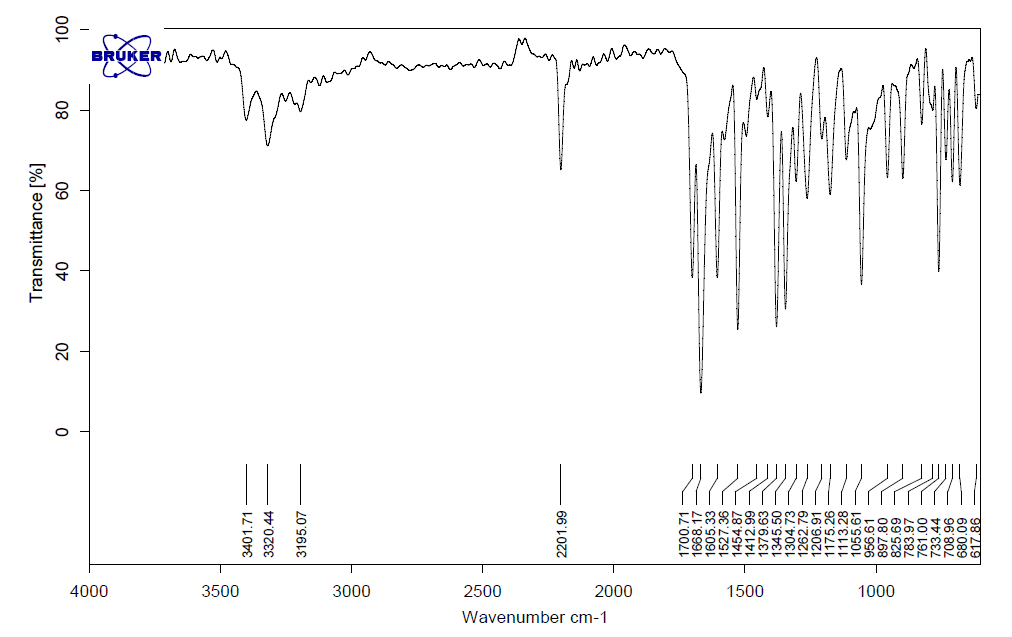


**The FT-IR of 2-Amino-4-(4-chlorophenyl)-5-oxo-4,5-dihydropyrano[3,2-*c*]chromene-3-carbonitrile**


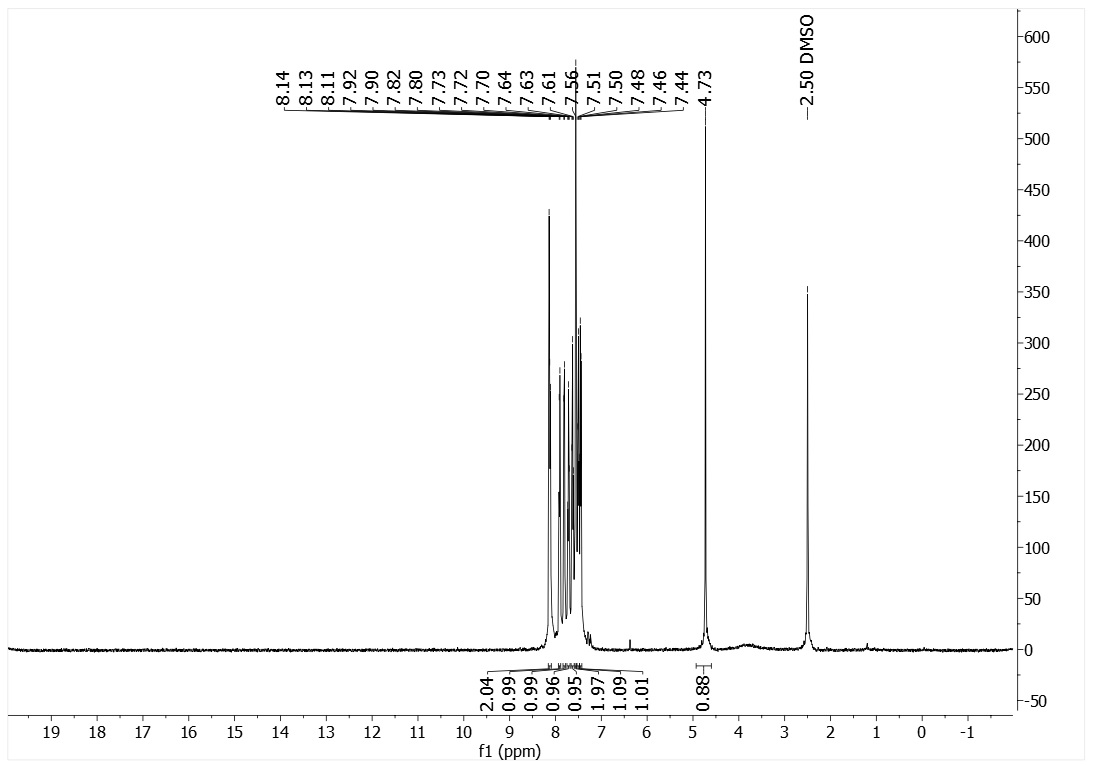


**The ^1^H NMR spectrum of 2-Amino-4-(4-chlorophenyl)-5-oxo-4,5-dihydropyrano[3,2-*c*]chromene-3-carbonitrile**


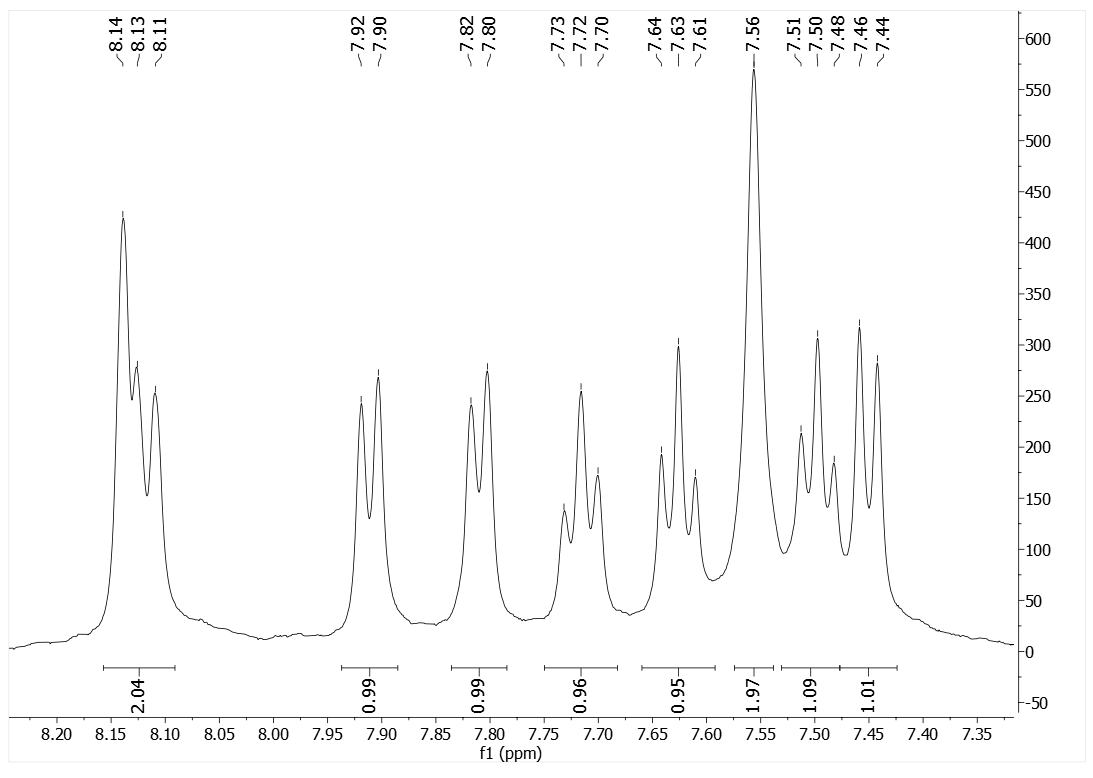


**The ^1^H NMR spectrum of 2-Amino-4-(4-chlorophenyl)-5-oxo-4,5-dihydropyrano[3,2-*c*]chromene-3-carbonitrile**


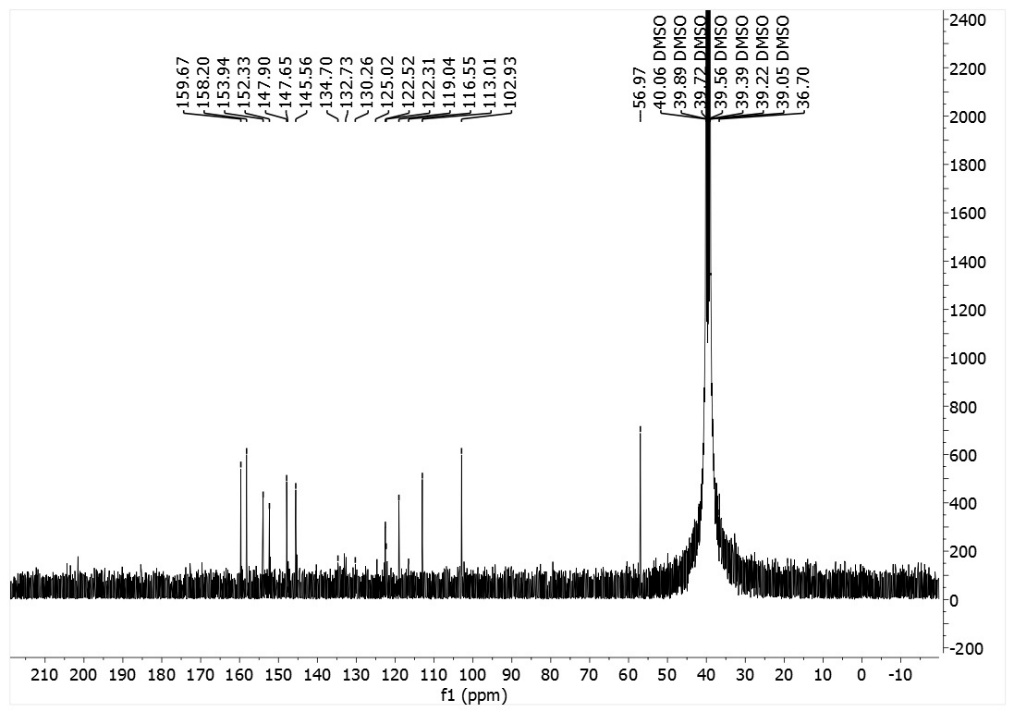


**The ^13^C NMR spectrum of 2-Amino-4-(4-chlorophenyl)-5-oxo-4,5-dihydropyrano[3,2-*c*]chromene-3-carbonitrile**
